# Dissection of the Influenza A Virus Endocytic Routes Reveals Macropinocytosis as an Alternative Entry Pathway

**DOI:** 10.1371/journal.ppat.1001329

**Published:** 2011-03-31

**Authors:** Erik de Vries, Donna M. Tscherne, Marleen J. Wienholts, Viviana Cobos-Jiménez, Florine Scholte, Adolfo García-Sastre, Peter J. M. Rottier, Cornelis A. M. de Haan

**Affiliations:** 1 Virology Division, Department of Infectious Diseases and Immunology, Faculty of Veterinary Medicine, Utrecht University, Utrecht, The Netherlands; 2 Department of Microbiology, Mount Sinai School of Medicine, New York, United States of America; 3 Department of Medicine, Division of Infectious Diseases, Mount Sinai School of Medicine, New York, United States of America; 4 Global Health and Emerging Pathogens Institute, Mount Sinai School of Medicine, New York, United States of America; Johns Hopkins University BSPH, United States of America

## Abstract

Influenza A virus (IAV) enters host cells upon binding of its hemagglutinin glycoprotein to sialylated host cell receptors. Whereas dynamin-dependent, clathrin-mediated endocytosis (CME) is generally considered as the IAV infection pathway, some observations suggest the occurrence of an as yet uncharacterized alternative entry route. By manipulating entry parameters we established experimental conditions that allow the separate analysis of dynamin-dependent and -independent entry of IAV. Whereas entry of IAV in phosphate-buffered saline could be completely inhibited by dynasore, a specific inhibitor of dynamin, a dynasore-insensitive entry pathway became functional in the presence of fetal calf serum. This finding was confirmed with the use of small interfering RNAs targeting dynamin-2. In the presence of serum, both IAV entry pathways were operational. Under these conditions entry could be fully blocked by combined treatment with dynasore and the amiloride derivative EIPA, the hallmark inhibitor of macropinocytosis, whereas either drug alone had no effect. The sensitivity of the dynamin-independent entry pathway to inhibitors or dominant-negative mutants affecting actomyosin dynamics as well as to a number of specific inhibitors of growth factor receptor tyrosine kinases and downstream effectors thereof all point to the involvement of macropinocytosis in IAV entry. Consistently, IAV particles and soluble FITC-dextran were shown to co-localize in cells in the same vesicles. Thus, in addition to the classical dynamin-dependent, clathrin-mediated endocytosis pathway, IAV enters host cells by a dynamin-independent route that has all the characteristics of macropinocytosis.

## Author Summary

Attachment to and entry into a host cell are the first crucial steps in establishing a successful virus infection and critical factors in determining host cell and species tropism. Influenza A virus (IAV) attaches to host cells by binding of its major surface protein, hemagglutinin, to sialic acids that are omnipresent on the glycolipids and glycoproteins exposed on the surfaces of cells. IAV subsequently enters cells of birds and a wide variety of mammals via receptor-mediated endocytosis using clathrin as well as via (an) alternative uncharacterized route(s). The elucidation of the endocytic pathways taken by IAV has been hampered by their apparent redundancy in establishing a productive infection. By manipulating the entry conditions we have established experimental settings that allow the separate analysis of dynamin-dependent (including clathrin-mediated endocytosis) and independent entry of IAV. Collectively, our results indicate macropinocytosis, the main route for the non-selective uptake of extracellular fluid by cells, as an alternative IAV entry route. As the dynamin-dependent and -independent IAV entry routes are redundant and independent, their separate manipulation was crucial for the identification and characterization of the alternative IAV entry route. A similar strategy might be applicable to the study of endocytic pathways taken by other viruses.

## Introduction

Influenza A virus (IAV) is an enveloped, segmented negative-strand RNA virus infecting a wide variety of birds and mammals. As its first step in infection IAV attaches to host cells by the binding of its major surface protein, the hemagglutinin (HA), to sialic acids, which are omnipresent on the glycolipids and glycoproteins exposed on the surfaces of cells. Where the structural requirements for this interaction have been studied in great detail, much less is known about whether and how the attachment to specific sialylated receptors (e.g. to N-linked glycoproteins, O-linked glycoproteins or gangliosides or even to specific receptors within these groups) affects the subsequent endocytic steps. Obviously, knowledge about the repertoire of endocytic pathways that can successfully be used by IAV will increase our insights into cell and species tropism of IAV. In turn, this will contribute to our understanding of the requirements for the generation of novel viruses with pandemic potential that can arise by exchange of RNA segments between currently circulating human serotypes and an animal virus during occasional co-infection in a human or an animal host.

Clathrin mediated endocytosis (CME) has for long been identified and studied as the major route of IAV cell entry [1], [2] and is, by far, the best characterized endocytic pathway. Evidence obtained from live cell imaging has revealed the *de novo* formation of clathrin-coated pits at the site of virus attachment [3] and the requirement for the adapter protein epsin 1, but not eps15, in this process [4]. Still, specific transmembrane receptors linking viral entry to epsin 1 or to other adapters have not been identified although a recent study performed in CHO cells indicated the specific requirement for N-linked glycoproteins in IAV entry [5].

Some recent papers provided indications for the utilization of alternative entry pathways by IAV. Studies in which CME was obstructed by pharmacological or genetic intervention indicated the ability of IAV to enter host cells via alternative endocytic routes [4], [6], [7]. Also live cell imaging revealed the simultaneous availability of entry routes involving non-coated as well as clathrin-coated pits [4]. However, this alternative IAV entry route has not been characterized in any detail and requirements for any specificity in receptor usage apart from the need for the proper sialic acid moiety have not been established.

During the past decades quite a variety of endocytic pathways have been identified in eukaryotic cells [8], [9], [10]. Their occurrence, abundance and mechanistic details appear to vary between cell types, tissues and species and their utilization by viruses as a route of entry makes them an important factor in host and cell-type permissiveness for infection [11], [12]. Besides by CME, different viruses have been shown to enter cells via caveolae, macropinocytosis or other, less well described, routes [11], [12]. Most often, the selection of a specific endocytic route is linked to the utilization of a specific receptor that facilitates traveling via that particular route. Nevertheless, many receptors allow flexibility by their capacity to enter through multiple pathways. For IAV, an additional level of complexity to the dissection of potential entry routes is added by the apparent lack of an IAV-specific protein receptor.

A full experimental characterization of the IAV entry pathways will benefit from separation of the IAV entry pathways into routes that can be studied independently. Whereas co-localization with clathrin is an established marker for endocytosis via this route, the complete lack of unique markers for macropinosomes or most other endocytic compartments [13], [14] complicates such studies. Furthermore, crucial to any study concerning endocytic pathways is the abundantly documented fact that such pathways are highly dependent on experimental cell culture conditions [15]–[19]. Pathways that are constitutive in one cell type may be absent or inducible by specific experimental conditions in other cell types. Moreover, the manipulation of specific endocytic pathways may result in up or down regulation of other specific pathways. Here we have established entry assay conditions that allow dissecting cell entry of IAV into a dynamin-dependent (DYNA-DEP) and a dynamin-independent (DYNA-IND) component. Dynamin is a large GTPase forming multimeric assemblies around the neck of newly formed endocytic vesicles. GTP hydrolysis is required for pinching off of the vesicles [20]. Whereas CME is completely dependent on dynamin, several other endocytic routes do not require dynamin [21]. We performed an extensive characterization of the dynamin-independent IAV entry route using pharmacological inhibitors as well as by expressing dominant-negative mutants and applying siRNA induced gene silencing as tools. Taken together the results identify a pathway that closely resembles macropinocytosis as a novel entry pathway for IAV.

## Results

### A luciferase reporter assay for quantitative analysis of IAV entry

To identify and characterize potential non-CME entry routes taken by IAV, we adapted a luciferase reporter assay [22] to enable the quantitative determination of infection or entry by measuring the activity of secreted Gaussia luciferase. Twentyfour hours prior to infection HeLa cells were transfected with a plasmid (pHH-Gluc) allowing constitutive synthesis (driven by the human PolI promoter) of a negative strand viral RNA (vRNA) encoding a Gaussia luciferase under control of the untranslated regions (UTRs) of the NP segment of Influenza A/WSN/33 (H1N1) (hereafter called IAV-WSN) NP segment. Upon IAV infection, the combined expression of the viral polymerase subunits and NP will drive transcription of luciferase mRNA from the negative strand vRNA and subsequent synthesis of Gaussia luciferase.

**Figure 1:**
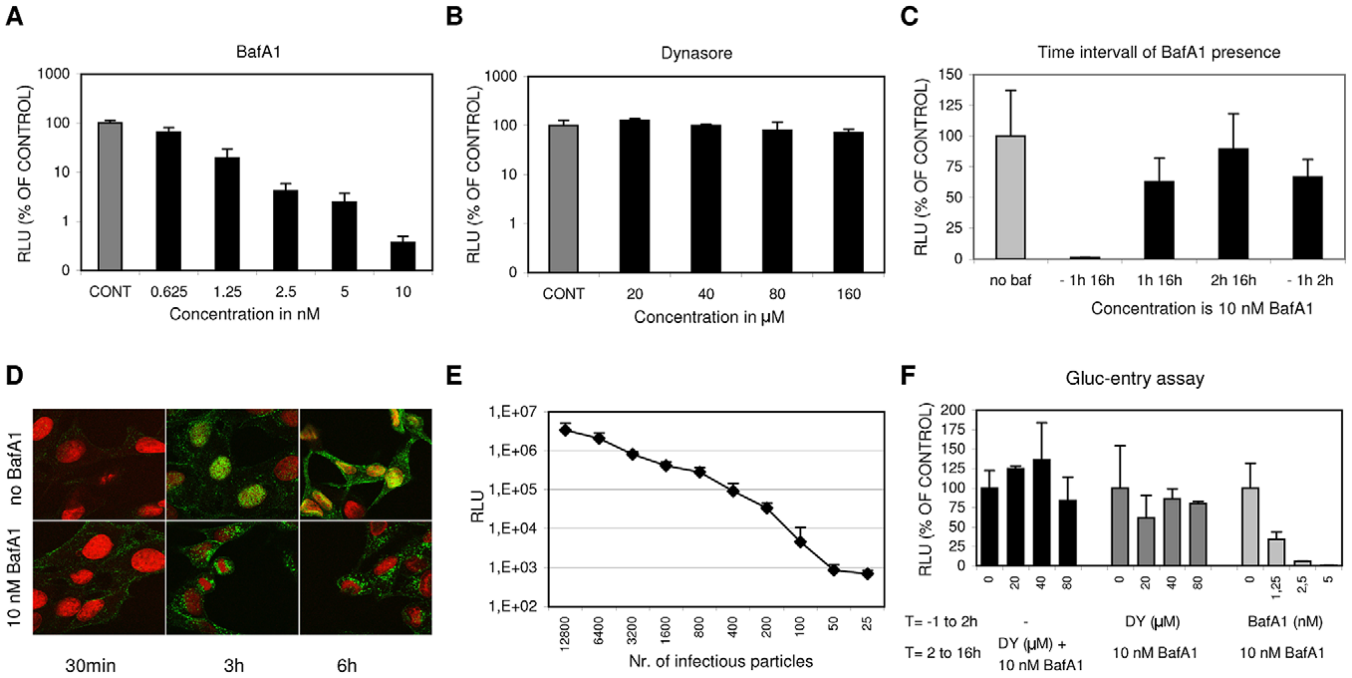
A luciferase reporter assay for quantitative analysis of IAV entry. (A, and B) HeLa cells were transfected with the IAV reporter plasmid pHH-Gluc 24 hrs before infection. Inhibitors were added at 1 hr before infection with IAV (strain WSN; MOI of 0.1). Cells were kept at 37°C in DMEM supplemented with 10% FCS at all times. Luciferase activity (RLU, Relative Light Units) was measured 16 hrs p.i. and plotted on the Y-axes relative to the control (infection in presence of 0.2% DMSO, the solvent of both inhibitors) (error bars represent S.D. derived from triplicates). (A) Inhibitory effect of BafA1 (concentration range 0.625 nM to 10 nM on the x-axis) (B) Inhibitory effect of Dynasore (concentration range 20 to 160 µM on the x-axis) (C) The effect of the presence of 10 nM BafA1 during different periods (−1 hr to 16 hrs p.i.; 1 hr to 16 hrs p.i.; 2 hrs to 16 hrs p.i. and −1 hr to 2 hrs p.i as plotted on the x-axis) of a multi-cycle infection with IAV (strain WSN; MOI 0.5). Y-axis and control as in panel A and B. BafA1 was only strongly inhibitory when already present before inoculation with IAV. Reversibility of inhibition was shown upon withdrawal of BafA1 at 2 hrs p.i. (−1 hr to 2 hrs). (D) Examination of the effect of 10 nM BafA1 on subcellular localization of IAV particles by confocal fluorescence microscopy. HeLa cells were grown on glass cover slips and infected with IAV (strain WSN; MOI of 10) and fixated after 30 min, 3 hrs or 6 hrs (column 1, 2 or 3 respectively). Infection was performed in 0.2% DMSO (upper row panels) or in the presence of 10 nM BafA1 (lower row panels). The nucleus was visualized by DNA staining with TOPRO-3 (red). IAV infection was visualized by staining with monoclonal antiserum directed against NP (green). In the absence of inhibitor, IAV localized to the nucleus after 3 hrs, while new virus particles spread to the cytoplasm after 6 hrs. BafA1 (lower row panels) caused accumulation of incoming virus particles at a peri-nuclear location. (E) Quantitative determination of IAV entry by a single-cycle Gluc-entry assay. HeLa cells (10,000 cells/well in DMEM supplemented with 10% FCS) were transfected with pHH-Gluc 24 hrs prior to infection with a serial dilution of infectious IAV particles (plotted on the x-axis). Two hours after infection 10 nM BafA1 was added to block any further entry. Cells were incubated for a further 14 hrs to allow expression of luciferase activity (y-axis; Relative Light Units, RLU). (F) Effect of Dynasore and BafA1 on IAV entry in the Gluc-entry assay. Dynasore (DY, dark grey bars; 20, 40 or 80 µM) or BafA1 (light grey bars; 1.25, 2.5 or 5 nM) were present from 1 hr prior to infection (strain WSN; MOI 0.5) to 2 hrs p.i. after which the inhibitor-containing medium was replaced with medium containing 10 nM BafA1 to block any further entry. Cells were incubated for a further 14 hrs to allow the quantitative expression of luciferase activity (y-axes; RLU relative to the control infection without inhibitor). Whereas BafA1 displayed dose-dependent inhibition of IAV entry, dynasore did not significantly inhibit IAV entry. Addition of Dynasore (DY, 20, 40 or 80 µM) at 2 hrs p.i. (black bars; MOI 0.5; no inhibitors present from −1 to 2 hrs) demonstrated that Dynasore does not have a post-entry effect.

A dose-response curve demonstrating the applicability of the assay to inhibitor screening (Fig. 1A) was obtained for Bafilomycin A1 (BafA1), a known inhibitor of IAV entry [23]. BafA1 acts upon the vacuolar-type H(+)-ATPase, thus preventing endosomal acidification and thereby trapping IAV in peri-nuclear immature endosomes with a lumenal pH that does not permit viral membrane fusion. Remarkably, dynasore, a small molecule inhibitor of the GTPase dynamin 2 that is crucial for endocytic vesicle formation in clathrin- and caveolin-mediated endocytosis [8] as well as in a poorly described clathrin- and caveolin-independent endocytic pathway [8], [19], did not give significant inhibition (Fig. 1B).

BafA1 specifically inhibits IAV during the entry phase as demonstrated in Fig. 1C. The continuous presence of 10 nM BafA1 (added to the cells 1 hr prior to infection) for 16 hrs completely prevents infection. In contrast the addition of BafA1 at 1 hr or 2 hrs post infection resulted in high levels of luciferase activity (again measured at 16 hrs p.i.) that were 63% or 90% respectively of the control to which no BafA1 was added, indicating that entry was essentially completed within 2 hrs. The last bar of Fig. 1C shows that the inhibition by BafA1 is reversible as withdrawal of the inhibitor after 2 hrs resulted in high levels of infection. The specific effect of BafA1 on IAV entry was confirmed by confocal microscopy demonstrating that BafA1, as expected, traps IAV particles in a peri-nuclear location, presumably in non-acidified endosomes (Fig. 1D). BafA1 was subsequently exploited to establish a specific IAV entry assay (hereafter further referred to as the Gluc-entry assay). HeLa cells transfected with pHH-Gluc were inoculated with IAV at a range of MOIs and incubated for 2 hrs after which the entry medium was replaced by complete growth medium containing 10% FCS and 10 nM BafA1 to prevent any further entry of virus. Entry was indirectly quantified by determination of luciferase activity after further incubation for 14 hrs demonstrating a quantitative correlation between infection dose and luciferase activity across a wide range of MOIs (Fig. 1E). The indirect Gluc-entry assay was next tested for its capacity to examine the effects of inhibitors on IAV entry. Dynasore or BafA1 (Fig. 1F) were included in the medium (DMEM containing 10% FCS) during entry (the first 2 h of infection) and were removed when the inoculum was replaced by growth medium containing BafA1. Concentrations up to 80 µM dynasore did not inhibit entry which is in agreement with the result shown in Fig. 1B. In contrast, 1.25 nM BafA1 already inhibited entry for more than 60% (Fig. 1F). As a control, dynasore was also added at 2 hrs post infection to analyze whether the drug affected IAV replication during the post entry phase. As expected, 80 µM dynasore did not significantly inhibit IAV replication when present from 2 to 16 hrs p.i. (Fig. 1F). Thus, with the Gluc-entry assay we can study the effect of specific inhibitors on IAV entry in a quantitative manner, at least as long as the inhibitors do not irreversibly affect IAV replication during the post entry phase.

Furthermore, the lack of inhibition of IAV entry by dynasore demonstrates that under these experimental conditions IAV is able to enter cells via a pathway that is fully redundant to any dynamin-dependent (DYNA-DEP) entry route, including the classical CME pathway. Also when IAV travels via this novel dynamin-independent (DYNA-IND) route, IAV apparently enters via low pH compartments as entry is fully sensitive to BafA1.

### Induction of a DYNA-IND IAV entry pathway by serum

**Figure 2:**
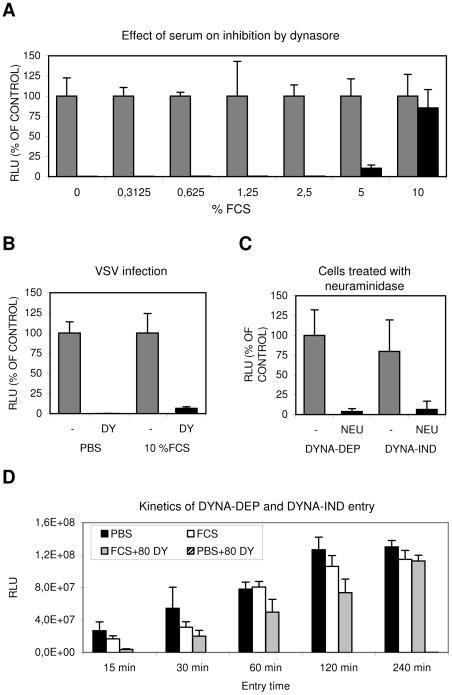
Serum induces a Dynasore resistant entry route. (A) The effect of increasing concentrations of FCS (in PBS) on IAV entry. HeLa cells were infected in the absence (grey bars) or presence (black bars) of 80 µM dynasore with IAV (strain WSN; MOI 0.5) using the Gluc-entry assay. Entry was determined in presence of increasing concentrations of FCS (plotted on the x-axis). Luciferase activity was determined 16 hrs p.i. (plotted on the y-axis relative to the activity obtained in absence of dynasore (grey bars, 100%) for each concentration of FCS. Entry of IAV became completely dynasore-resistant in the presence of 10% FCS. (B) Inhibition of VSV entry by 80 µM dynasore (DY) in presence of 10% FCS. HeLa cells were infected with VSV in PBS or PBS+10% FCS (labeled 10% FCS) in absence (grey bars) or presence (black bars) of 80 µM dynasore (DY). In presence of 10% FCS VSV infection appears still to be fully sensitive to dynasore. (C) IAV entry via the dynamin-dependent (DYNA-DEP) or dynamin-independent (DYNA-IND) route depends on the presence of sialic acid receptors. Hela cells were treated with *V. cholerae* neuraminidase (NEU; black bars) or mock-treated (-; grey bars) prior to infection in order to remove surface exposed sialic acids. Cells were infected (strain WSN; MOI 0,5) using the Gluc-entry assay. DYNA-DEP entry was determined in PBS and DYNA-IND entry in PBS containing 10% FCS and 80 µM dynasore. Both pathways appear to be equally sensitive to de-sialylation of cells. (D) Comparison of the kinetics of DYNA-DEP IAV entry and DYNA-IND IAV entry. DYNA-DEP entry was determined in PBS and DYNA-IND entry in PBS containing 10% FCS and 80 µM dynasore (80 DY) using the Gluc-entry assay as described for panel C. Entry was allowed to proceed for 15 to 240 minutes (x-axis) by the addition of full growth medium containing 10 nM BafA1 at the indicated timepoints p.i. Luciferase activity was plotted in absolute RLU (y-axis), demonstrating that similar entry efficiencies were obtained via the two pathways after 4 hrs of entry. The experiment was repeated several times with different batches of serum yielding similar results.

As factors present in serum are known for their potential to induce specific endocytic pathways, we further explored the conditions required for the novel DYNA-IND IAV entry pathway (using the Gluc-entry assay) by inoculating cells in PBS in the presence of increasing concentrations of fetal calf serum (FCS). Whereas dynasore completely inhibited entry in PBS, inclusion of 5% and 10% FCS resulted in increasing levels of dynasore resistant entry (Fig. 2A), suggesting the existence of a serum-inducible DYNA-IND IAV entry pathway. This effect was not caused by inactivation of dynasore during the experiment as vesicular stomatitis virus (VSV), which enters cells by CME [24], [25], was still sensitive to 80 µM dynasore in the presence of 10% FCS (Fig. 2B). In agreement herewith, the uptake of transferin, known to occur via CME, was inhibited by dynasore regardless of the presence of FCS (Fig. S2, panel A). As expected, both DYNA-DEP entry in PBS and DYNA-IND entry in the presence of 10% FCS and 80 µM dynasore required sialic acid receptors for efficient entry as pre-treatment of HeLa cells with neuraminidases almost completely abolished entry via either pathway (Fig. 2C). The kinetics of the DYNA-DEP and DYNA-IND entry pathways were compared by performing a time-course experiment in which IAV entry was terminated by the addition of 10 nM BafA1 at different time points (Fig. 2D). In comparison to entry via the DYNA-DEP pathway (the only pathway available in PBS) entry in the presence of FCS (when presumably both the DYNA-DEP and DYNA-IND entry pathways are available) showed similar kinetics. In contrast, entry via the DYNA-IND pathway (which is the only pathway that is active in the presence of 10% FCS and 80 µM dynasore) was slower. The difference was most prominent after 15 min, while after 4 hrs similar levels of entry were reached.

**Figure 3:**
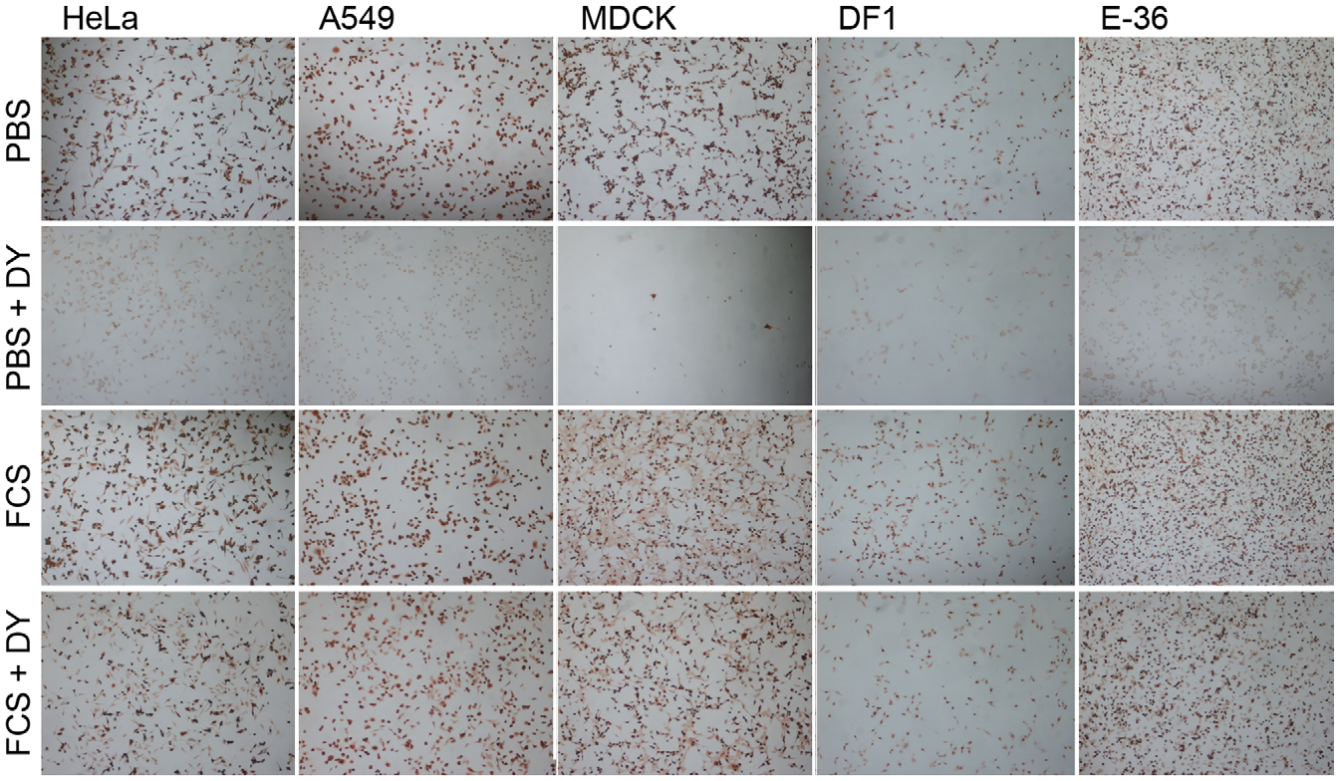
Serum-inducible DYNA-IND IAV entry in different cell types. HeLa cells, A549 cells (human epithelial lung carcinoma cells), MDCK cells (canine kidney cells), DF-1 cells (chicken embryonic fibroblast cells) and E-36 cells (Chinese hamster lung cells) were infected with IAV (MOI 2) in PBS or in PBS supplemented with 10% FCS in the absence or presence of 80 µM dynasore (DY). After 2 hrs the entry medium was replaced by growth medium (DMEM with 10% FCS and 10 nM BafA1) and infection was continued for 14 h after which the cells were fixed, permeabilized and stained with a monoclonal antibody against NP (detection by peroxidase staining).

To validate and extend these results we visualized the reduction of the number of infected cells by immunoperoxidase staining using an antibody against NP (Fig. 3). A number of different cells of mammalian and avian origin were infected for 2 hours at an MOI of 1 in PBS with or without serum. After 2 hours the inoculum was replaced by growth medium containing 10% FCS and 10 nM BafA1 and the expression of NP was examined after 14 hours later. After incubation in PBS, staining was completely prohibited by the presence of 80 µM dynasore whereas in the presence of serum dynasore had no effect. A serum-inducible, DYNA-IND route of entry was thus functional in all five cell lines, including the human epithelial airway carcinoma cell line A549.

**Figure 4:**
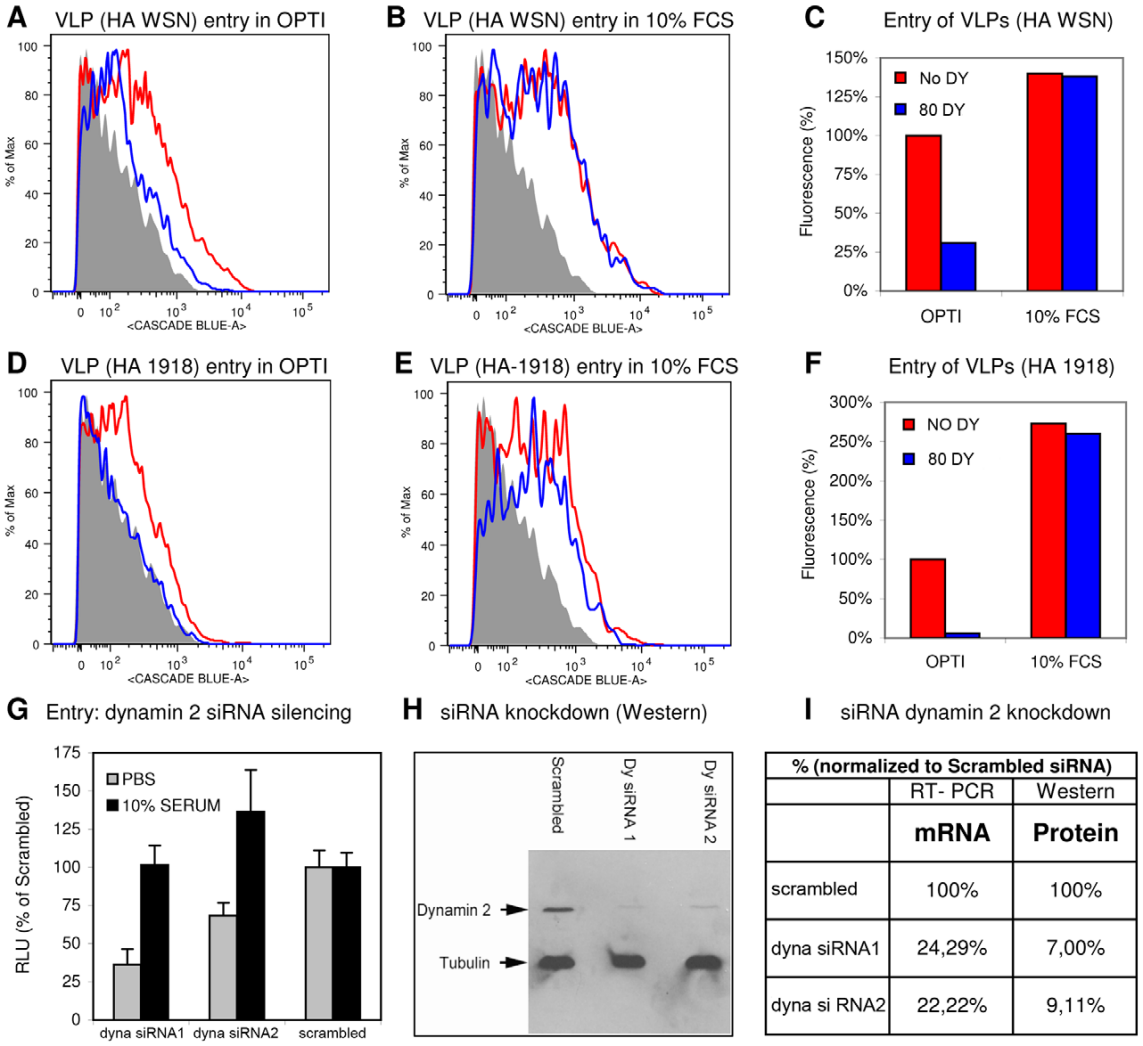
Identification of DYNA-IND entry in a VLP entry assay and by siRNA knockdown of dynamin 2. FACS histograms displaying the entry of IAV VLPs, containing the BlaM1 reporter and carrying either the WSN (A and B) or the 1918 (D and E) HA and NA, in Optimem (OPTI) without (A and D) or with (B and E) 10% FCS, and in the absence (red lines) or presence (blue lines) of 80 µM dynasore are shown. Release of BlaM1 into the cytoplasm upon VLP fusion results in cleavage of the CCF2 substrate thereby causing emission of a fluorescent signal at 447 nm (X-axis; Cascade Blue). In the histograms entry is displayed by a shift to higher fluorescence (the grey area represents background fluorescence of non-infected cells). (C and F) Quantification of FACS results. Background fluorescence was subtracted from each measurement (geometric mean) and data were normalized to VLP entry in Optimem without dynasore (DY) (red curve of panel A and and D). VLP entry was not inhibited by dynasore in presence of 10% FCS whereas the access of BlaM1 to its CCF2 substrate in the cytoplasm was blocked by dynasore in PBS. VLP entry was more efficient in the presence of serum. (G) Effect of downregulation of dynamin 2 by siRNA silencing. Serum-inducible DYNA-IND entry was analyzed in HeLa cells that were transfected 48 hrs prior to infection with two different siRNAs targeting dynamin-2 (dyna). siRNA treated cells were infected with the pseudovirus WSN-Ren in PBS (grey bars) or in PBS containing 10% FCS (black bars) and luciferase activity was determined after 16 hrs post infection (y-axis; RLU relative to infection of cells transfected with a scrambled siRNA). Entry of pseudovirus WSN-Ren (MOI 0.5) was reduced by 50% to 70% when entry was performed in PBS (grey bars) whereas entry was not significantly affected in the presence of 10% serum (black bars). (H) Western blot showing the knockdown of dynamin 2 (in comparison to tubulin) at 48 hrs after transfection with siRNAs. (I) Quantification of the residual levels of dynamin 2 (dyna) mRNA (determined by quantitative RT-PCR) and protein (determined by densitometric scanning of the western blot) 48 hrs after siRNA transfection. Data were normalized to 18S RNA (RT-PCR) or tubulin protein levels and calculated relative to the levels obtained after transfection with a scrambled siRNA that served as a control.

To confirm our results and to obtain further proof for the utilization of DYNA-DEP and DYNA-IND entry routes by IAV, we additionally used an IAV virus-like particle (VLP) direct entry assay [26]. These VLPs contain IAV HA and NA in their envelope and harbor a beta-lactamase reporter protein fused to the influenza matrix protein-1 (BlaM1), which allows the rapid and direct detection of entry, independent of virus replication. Upon fusion of viral and endosomal membrane, BlaM1 gains access to the cytoplasmically retained fluorigenic substrate CCF-2 that, after cleavage by BlaM1, shifts to a shorter fluorescent emission wavelength that can be detected by flow cytometry. Entry into HeLa cells was performed in the absence or presence of 10% FCS using VLPs containing HA and NA either from IAV-WSN (having a strict alpha 2–3 linked sialic acid binding specificity) or from the pandemic 1918 IAV (HA from A/NewYork/1/18, binding to alpha 2–3 and alpha 2–6 linked sialic acids; NA from A/BrevigMission/1/18). Entry of VLPs of both IAV strains was severely inhibited by dynasore when no serum was added to the inoculum (Fig. 4A, 4D), whereas the presence of 10% FCS rendered entry completely dynasore resistant. (Fig. 4B, 4E). Quantification of VLP entry is shown in Fig. 4C and F.

Importantly, to confirm the existence of the serum-inducible entry pathway by a method that is independent of dynasore, we used siRNA induced silencing of dynamin 2. Fig. 4G shows that two different siRNAs had a significant inhibitory effect (48 hrs after siRNA transfection) on entry of the *Renilla* luciferase-encoding pseudovirus WSN-Ren [27] in HeLa cells in the absence of FCS, whereas the presence of 10% serum no reduction in entry levels was observed, confirming the results obtained with dynasore. Knockdown of dynamin 2 protein levels (48 hrs after siRNA transfection) was analyzed by western blotting (Fig. 4H) and quantified in Fig. 4I which also shows the knockdown of dynamin 2 mRNA levels as determined by quantitative RT-PCR.

We conclude that a DYNA-IND entry pathway can be induced by serum in different cell types from several species. The evidence was obtained using both replication-dependent (Gluc-entry assay and immunodetection of infected cells) and replication-independent assays (entry of VLPs), the latter allowing immediate detection of the fusion-mediated delivery of viral M1 protein into the cytoplasm.

### Inhibitors of growth factor receptor tyrosine kinases and actomyosin network dynamics reduce DYNA-IND entry of IAV

**Figure 5:**
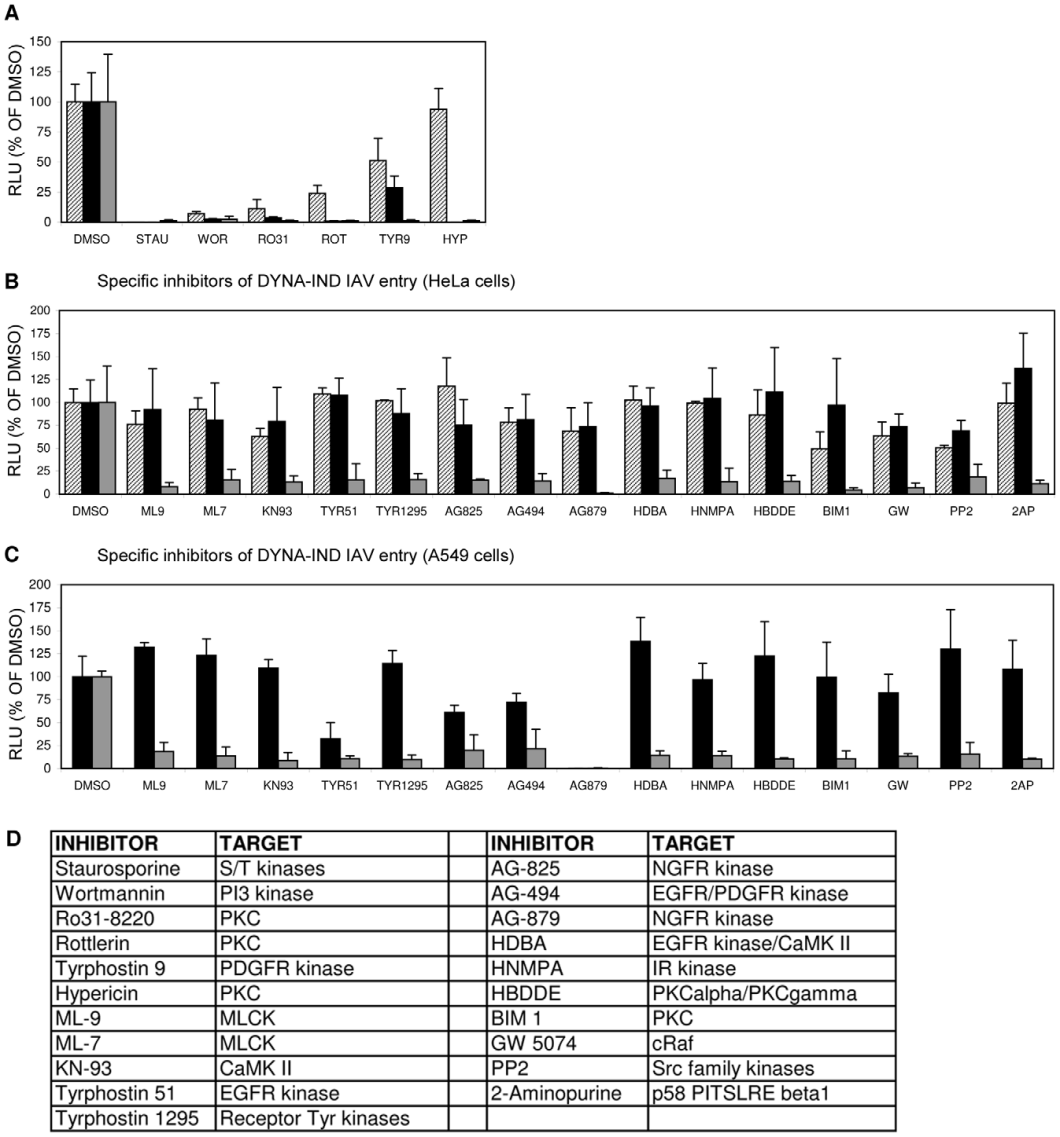
Screening of an eighty-compound protein kinase inhibitor library in an IAV entry assay. HeLa cells were incubated with kinase inhibitors at a concentration of 10 µM from 1 hr prior to infection (strain WSN; MOI 0.5). Entry (Gluc-entry assay) was performed for 2 hrs in the presence of the inhibitors under DYNA-DEP entry conditions (entry in PBS; black bars) and DYNA-IND entry conditions (entry in PBS supplemented with 10% FCS and 80 µM dynasore; grey bars. In addition, all inhibitors were added 2 hrs p.i. to cells that had not been treated with inhibitors during entry (in presence of 10% FCS) in order to screen for post-entry effects (striped bars). (A) Inhibitors that were inhibitory for both entry routes and mostly also affected post-entry events. (B) Inhibitors that affect DYNA-IND entry but neither displayed effects on DYNA-DEP entry or post-entry effects. (C) All kinase inhibitors were also screened on A549 cells. Black and grey bars correspond to DYNA-DEP and DYNA-IND entry. (D) Targets of inhibition of the different kinase inhibitors are listed. All inhibitors have been tested at a standard concentration of 10 µM. Whereas for some inhibitors higher concentrations may be required for efficient inhibition of their specific target, for other inhibitors this concentration is relatively high and other targets may be inhibited as well. For instance wortmannin is known to inhibit PI4 kinase and MLCK at a 10 µM concentration.

The DYNA-IND entry pathway was further characterized by inhibitor profiling using an 80-compound kinase inhibitor library. Serum-induced DYNA-IND entry was examined in 10% FCS using the Gluc-entry assay. 80 µM dynasore was added in order to block CME and any other potential DYNA-DEP entry pathways. This allowed the independent inhibitor profiling of the novel pathway by avoiding the potentially masking effect of the presence of redundant entry pathways. Cells were preincubated with the kinase inhibitors (10 µM) for 1 h at 37°C and then inoculated with virus (MOI 0.5) in the presence of 10% FCS and 80 µM dynasore for 2 h at 37°C (DYNA-IND entry). In parallel, inoculations were also done in PBS to compare the effects of the inhibitors on DYNA-DEP entry. After 2 hr the medium and inhibitor were replaced by full growth medium containing 10% FCS and 10 nM BafA1 to allow the subsequent expression of Gluc activity under identical conditions for the DYNA-IND and -dependent entry assay. Six kinase inhibitors appeared to act non-discriminatively, inhibiting both DYNA-DEP and DYNA-IND entry (Fig. 5A): the protein kinase C (PKC) inhibitors Ro 31-8220, rottlerin (both displaying moderate cytotoxicity, result not shown) and hypericin, which have all three been previously identified as IAV inhibitors [28], [29]; the highly cytotoxic pan-specific serine/threonine protease inhibitor staurosporine; the irreversible PI-3 kinase inhibitor wortmannin and the receptor tyrosine kinase inhibitor TYR9. In order to investigate whether some of these inhibitors affect IAV replication during the post-entry phase, we performed the same experiments but now adding the kinase inhibitors after viral entry. Four of the inhibitors thus appeared to induce significant inhibition of post-entry processes (Fig. 5A). Although unlikely, we cannot formally exclude that post-entry processes specific for only one of the two entry pathways are affected. Interestingly, whereas no specific DYNA-DEP entry inhibitors were identified, 15 inhibitors (none displaying cytotoxic effects, data not shown) caused significant (p<0.05) inhibition (>5-fold) of DYNA-IND entry (Fig. 5B). This included inhibitors of the calmodulin dependent kinases myosin light chain kinase (MLCK) and CaMKII and seven inhibitors of different growth factor receptor tyrosine kinases. In contrast to the three non-specific PKC inhibitors mentioned above, the PKC inhibitors BIM-1 and HBDDE appeared to have a specific inhibitory effect on DYNA-IND entry. The specific effect of these drugs on DYNA-IND entry is not only shown by the lack of inhibition of DYNA-DEP entry in PBS, but also by the observation that none of the fifteen compounds induced >2-fold inhibition when added post-entry (at t = 2 hr post infection). The kinase library screen was repeated on A549 human epithelial lung carcinoma cells in order to confirm the results in a potentially more natural host cell line. The inhibition profiles obtained were very similar to those found for HeLa cells with the exception of the strong effect of AG879 (99% inhibition) and moderate effects of AG825 (39% inhibition) and Tyr51 (68% inhibition) on DYNA-DEP entry. (Fig. 5C).

**Figure 6:**
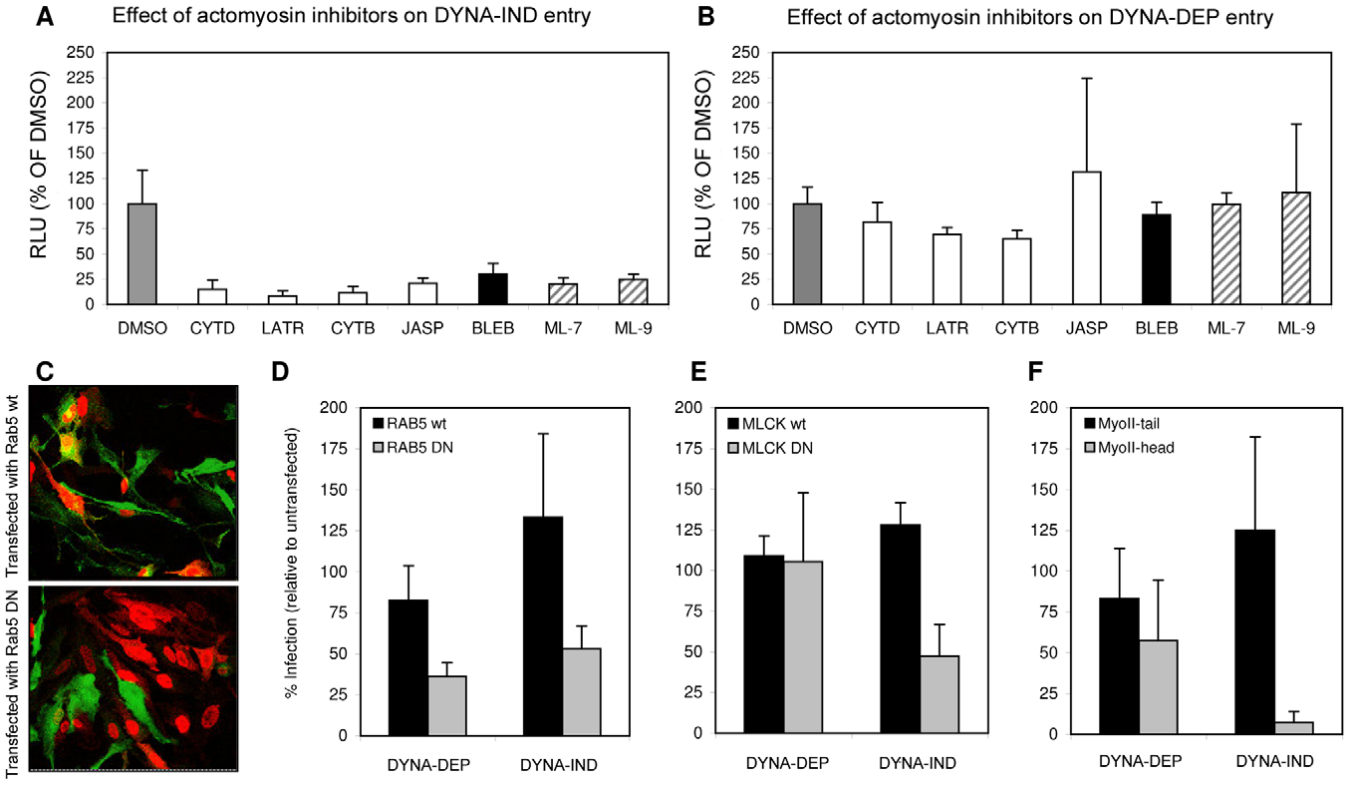
Actomyosin network dynamics are required for DYNA-IND entry by IAV. (A and B) Compounds affecting actin polymerization/de-polymerization (20 µM cytochalasin D [CYTD], 20 µM cytochalasin B [CYTB], 1 µM Latrunculin A [LATR], 1 µM Jasplakinolide [JASP]), inhibitors of MLCK (5 µM ML-7 and 5 µM ML-9) and an inhibitor of myosin II, (80 µM Blebbistatin [BLEB]) were examined for their effect on DYNA-IND IAV entry in PBS (A) or DYNA-DEP IAV entry (in PBS, 10% FCS and 80 µM dynasor) (B) using the Gluc-entry assay (HeLa cells; strain WSN; MOI 0.5; incubation with inhibitors from 1 hr prior to infection to 2 hrs p.i.). Luciferase activity was determined 16 hrs p.i. (RLU plotted on the y-axis relative to the activity obtained in absence of compounds affecting the actomysoin network (grey bars, 100%). (C) An example of confocal images of HeLa cells, expressing a wildtype Rab5-GFP fusion protein (Rab5 wt) (upper panel, green fluorescence identifies transfected cells) or a dominant-negative mutant of a Rab5-GFP fusion protein (RAB5 DN; lower panel), inoculated with IAV (strain WSN; MOI 1). Infection was performed for 4 hrs after which cells were fixed and stained (red staining with a monoclonal antibody directed against NP identifies infected cells; In the example of panel C infection was performed in PBS in order to examine the DYNA-DEP entry pathway) Similar experiments were performed for GFP fusion proteins with dominant-negative and wildtype MLCK (MLCK DN and MLCK wt) and Myosin II-tail domain (MyoII-tail) or MyosinII-head domain (MyoII-head). Transfected cells were infected under DYNA-DEP (PBS) and DYNA-IND (PBS, 10% FCS and 80 µM dynasor) entry conditions. (D–F) Results were quantified by counting >100 cells (experiment performed in triplo; transfected cells, infected cells and cells that were transfected as well as infected were counted). Relative infection of transfected cells was plotted (taking infection of non-transfected cells in the same sample as 100%). Thus, whereas Rab5 wt transfected cells are not significantly reduced or enhanced in infection compared to non-transfected cells (D, black bars), Rab5 DN transfected cells (D, grey bars) are infected at lower levels both under DYNA-DEP and DYNA-IND entry conditions. In contrast, cells transfected with MCLK DN (E) or MyoII-head (F) were infected at significantly lower levels via the DYNA-IND pathway while the DYNA-DEP pathway was not affected. Transfection efficiency for the different constructs was as follows: RAB5 wt (61%); RAB5 DN (45%); MLCK wt (50%); MLCK DN (49%); MyoII-tail (69%); MyoII-head (55%).

MLCK inhibitors ML-7 and ML-9 have been reported to be highly specific for their target kinase [30]. Phosphorylation by MLCK activates non-muscle myosin II light chain, indicating that a functional actomyosin network might be essential for DYNA-IND entry of IAV. This was further examined by testing the effect of Blebbistatin, an inhibitor of myosin II heavy chain activity, and of several inhibitors that affect actin dynamics by disrupting actin microfilaments (Cytochalasin B and D), by enhancing actin polymerization (Jasplakinolide) or by inhibiting actin polymerization (Latrunculin A). Actin inhibitors were used at the minimal concentration required to induce clearly visible changes in the actin cytoskeleton as pre-determined by staining with FITC-phalloidin (results not shown). Whereas the inhibitors did not affect DYNA-DEP entry (Fig. 6A) using Gluc-entry assay, all inhibitors as well as ML-7 and ML-9 significantly inhibited DYNA-IND entry (Fig. 6B).

Next, HeLa cells were transfected with plasmids encoding dominant negative or wildtype Rab5 fused to green fluorescent protein (Rab5 DN and Rab5 wt in Fig. 6) 24 h prior to infection with IAV. Rab5 is a small GTPase found in association with several endosomal compartments and crucial for the function and maturation of early endosomes. It is required for the trafficking of a wide range of endocytic cargo following different routes, including DYNA-DEP as well as DYNA-IND routes [31]. Entry of IAV has been shown to require Rab5 [32]. Consistently, we found that HeLa cells expressing Rab5 DN (as identified by GFP fluorescence, Fig. 6C) were much less susceptible to productive IAV infection (as judged by indirect immunofluorescence using Alexa-488 labeled NP antibodies) than cells transfected with Rab5 wt, both by DYNA-DEP (64% inhibition) and by DYNA-IND (47% inhibition) routes (Fig. 6D). In contrast, when examining the efficiency of DYNA-DEP and DYNA-IND entry routes in cells transfected with a dominant negative mutant of MLCK (MLCK DN; examined in comparison to MLCK wt), only IAV infection under DYNA-IND conditions was significantly reduced (Fig. 6E; 53% inhibition). Similarly, a construct encoding the N-terminal head domain of myosin II MyoII-head) also only significantly affected DYNA-IND entry (Fig. 6F; 92% inhibition). The combined results indicate that a dynamic actomyosin network requiring the activation of myosin II by MLCK is necessary for efficient entry of IAV via a DYNA-IND pathway.

### DYNA-IND IAV entry follows a pathway with similarities to macropinocytosis

**Figure 7:**
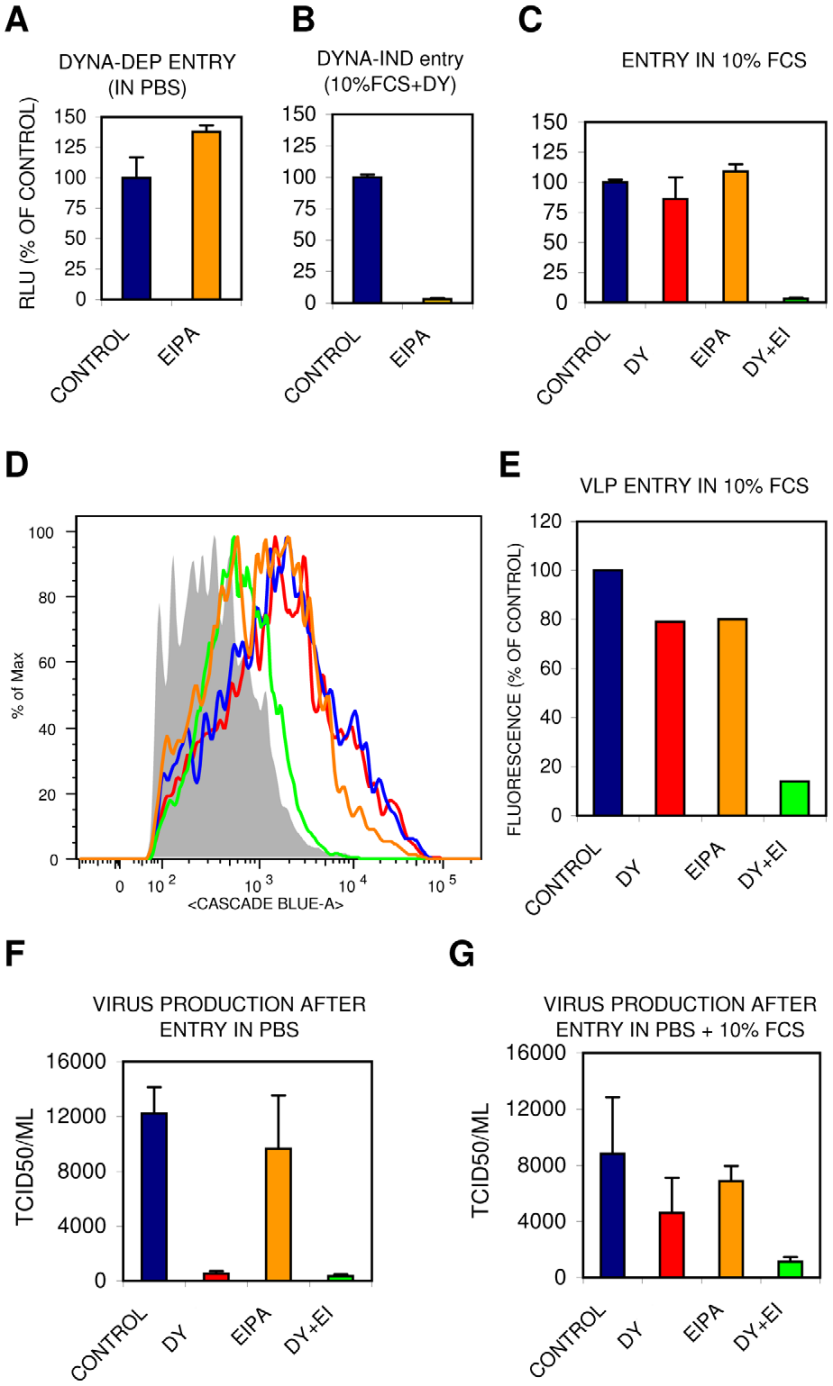
The DYNA-IND IAV entry pathway is EIPA sensitive. The effect of 80 µM EIPA on DYNA-DEP (A) or DYNA-IND (B) entry was examined in the Gluc-entry assay (HeLa cells; strain WSN; MOI 0.5; incubation with EIPA from −1 hr to 2 hr p.i.). Data were plotted relative to the control (0.2% DMSO). (C) Redundancy of DYNA-DEP and DYNA-IND entry pathways in 10% FCS using the Gluc-entry assay. The effect of 80 µM dynasore (DY; red), 80 µM EIPA (orange) or 80 µM of both inhibitors (DY+EI; green) in the presence of 10% FCS is shown (HeLa cells; strain WSN; MOI 0.5). (D,E) Redundancy of DYNA-DEP and DYNA-IND entry pathways in 10% FCS using the VLP entry assay. VLP entry was performed in Optimem containing 10% serum in the presence of 80 µM dynasore (DY; red), 80 µM EIPA (orange) or 80 µM of both inhibitors (DY+EI; green). Panel E displays the quantified data of the FACS histograms displayed in panel D. (F,G) Virus production was measured by determining infectivity in the supernatant of cells inoculated in the presence of 80 µM dynasore (DY), 80 µM EIPA or 80 µM of both inhibitors (DY+EI) in PBS (panel E) or PBS+10% FCS (panel F). After 2 hr the medium was replaced by growth medium containing 10% FCS and 10 nM BafA1. Supernatant was harvested 24 hr p.i. for TCID50 determination (Y-axis).

Several dynamin-independent endocytic pathways have been described [8], [19]. Of these, macropinocytosis has been demonstrated to be stimulated by growth factors present in serum and to depend on actin dynamics [12]–[14]. Yet, studies on macropinocytosis are hampered by a lack of specific inhibitors, cargo, membrane markers and characteristic morphology. Amiloride and the more potent derivative EIPA are inhibitors of epithelial sodium channels (ENaC) as well as of several other Na+/H+ antiporters. EIPA has often been used as a hallmark inhibitor that specifically inhibits endocytosis via the macropinocytic pathway [14]. Whereas DYNA-DEP entry of IAV was not inhibited by EIPA (Fig. 7A), DYNA-IND entry was fully blocked EIPA (Fig. 7B). The existence of redundant entry pathways in the presence of 10% FCS is clearly demonstrated by the marginal inhibition by either EIPA or dynasore whereas the combination of EIPA and dynasore resulted in strong inhibition both in the Gluc-entry assay (Fig. 7C) and in the direct VLP entry assay (Fig. 7D and E). Supplementary Fig. S1 shows that other cell lines, including the human lung epithelial cell line A549, display similar IAV inhibition patterns for EIPA and dynasore. Consistently, virus production displayed a similar inhibitor sensitivity profile (Fig.7 F and G) as virus entry indicating that the entry pathways we characterized lead to a productive infection. Clearly, VLPs and viral particles follow similar redundant entry pathways, distinguishable in a DYNA-DEP and a DYNA-IND pathway, the latter being sensitive to EIPA and dependent on actomyosin function.

**Figure 8:**
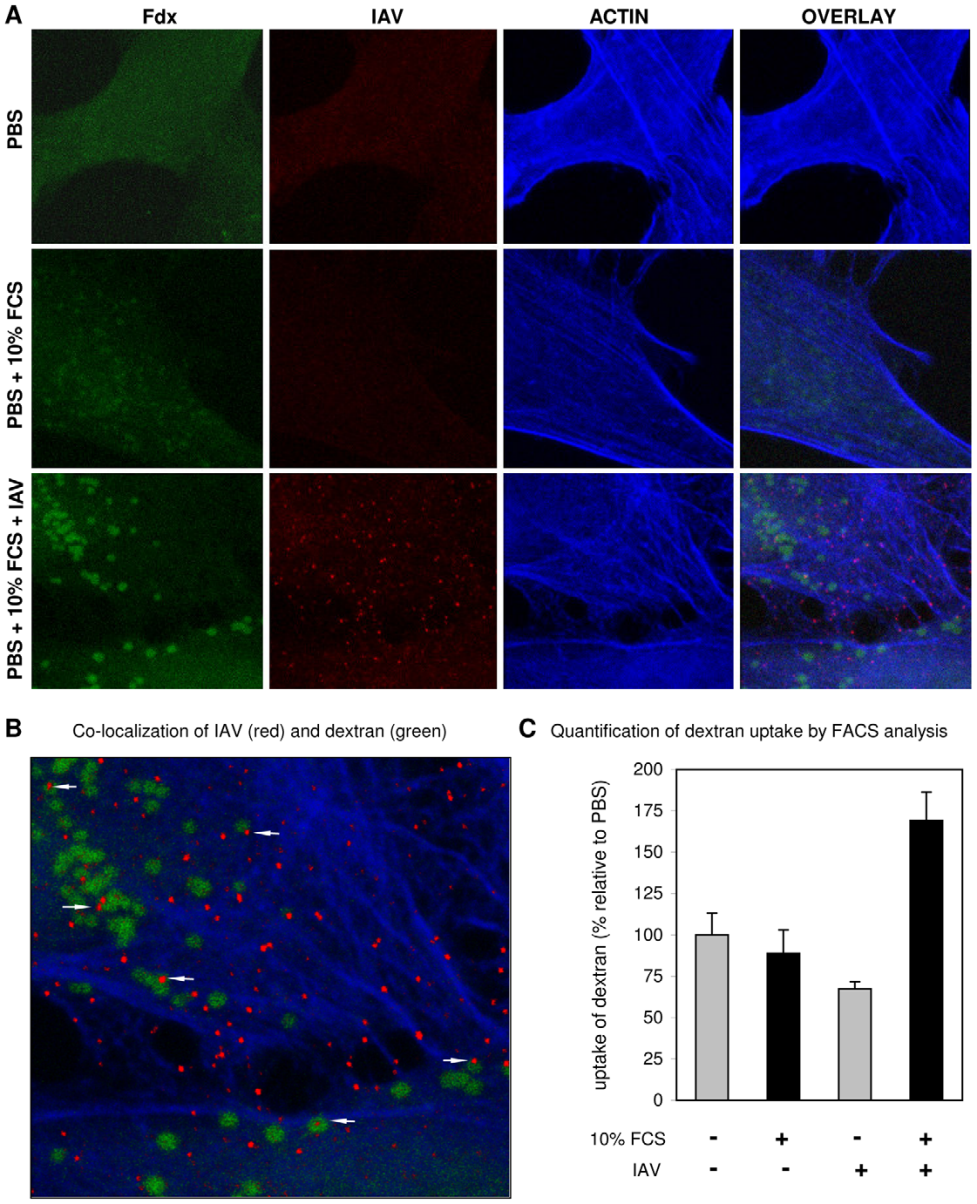
Induction of uptake of dextran into vesicles by the combined action of serum and IAV. (A) Uptake of FITC-dextran (Fdx; green) into HeLa cells in PBS, PBS+10% serum or PBS+10% serum+IAV (MOI = 10) was studied by confocal microscopy. IAV was detected by fluorescence staining with monoclonal antibody directed against NP. The actin network of cells was stained by phalloidin. Whereas vesicular structures containing Fdx become visible in the presence of serum, these structures become much more apparent in the simultaneous presence of serum and IAV. (B) A magnification of Panel A demonstrating the co-localization of Fdx and IAV in vesicular structures (arrows). (C) Quantification of dextran uptake in presence or absence of IAV by FACS analysis. Fdx uptake was performed for 15 min at 37°C in PBS (grey bars) or in PBS containing 10% FCS (black bars) in absence or presence of IAV (strain WSN; MOI 10) as indicated at the x-axis. Background fluorescence from Fdx binding to the outside of cells was determined by performing the same experiment at 4°C (at which no endocytosis takes place) and was subtracted from the mean fluorescence intensity obtained at 37°C to determine the amount of fluorescent FITC-dextran that was internalized at 37°C. Data were plotted relative to FITC-dextran uptake in PBS in absence of IAV.

One characteristic of macropinocytosis is the nonselective uptake of large amounts of extracellular solutes [33]. Therefore, the uptake of soluble FITC labeled dextran (Fdx) into relatively large vesicles (0.3 to 5 µM) has often been applied as a morphological marker for macropinosomes. Using this marker we found that the addition of 10% FCS to the culture medium slightly increased the uptake of Fdx into HeLa cells (Fig. 8A). Notably, the distribution of Fdx changes in response to serum from a random distribution into a more granular pattern. At high magnification and at color settings adjusted to higher intensity it could be seen that these Fdx granules were free of actin staining (by phalloidin) indicating that they were in the lumen of vesicles (result not shown). Interestingly, in the presence of IAV (MOI of 10) the uptake of Fdx into vesicles was clearly enhanced. At a higher magnification viral particles could be found to co-localize in Fdx loaded vesicles as well as outside these vesicles (Fig. 8B). Phalloidin staining of actin was used to demonstrate that many virus particles localized to actin-rich protrusions at the periphery of the cell. The uptake of Fdx was studied in a quantitative manner by flow cytometry (Fig. 8 C). A moderate, but reproducible shift to higher Fdx fluorescence was observed at 37°C when virus was added in presence of 10% FCS whereas such a shift was absent when no serum or virus was added. This result confirms the observations by confocal microscopy (Fig. 8A) which showed that the combined presence of FCS and IAV increases the uptake of Fdx as compared to FCS alone. In a control experiment the uptake of Fdx in 10% FCS in presence of IAV was shown to be specifically inhibited by EIPA, but not by dynasore (Fig. S2, panel B). In contrast, transferrin uptake, which serves as a specific marker for CME, was affected by dynasore, but not by EIPA (Fig. S2, panel A).

In conclusion, serum induces the uptake of Fdx into large vesicles, which can be further enhanced by the addition of IAV particles that, after entry, co-localize in part with these vesicles. These results indicate the utilization of a macropinocytic pathway for entry of IAV, which is consistent with the observed sensitivity of the serum-inducible DYNA-IND entry of IAV and VLPs to EIPA.

### IAV entry via macropinocytosis depends on PAK1 and src

**Figure 9:**
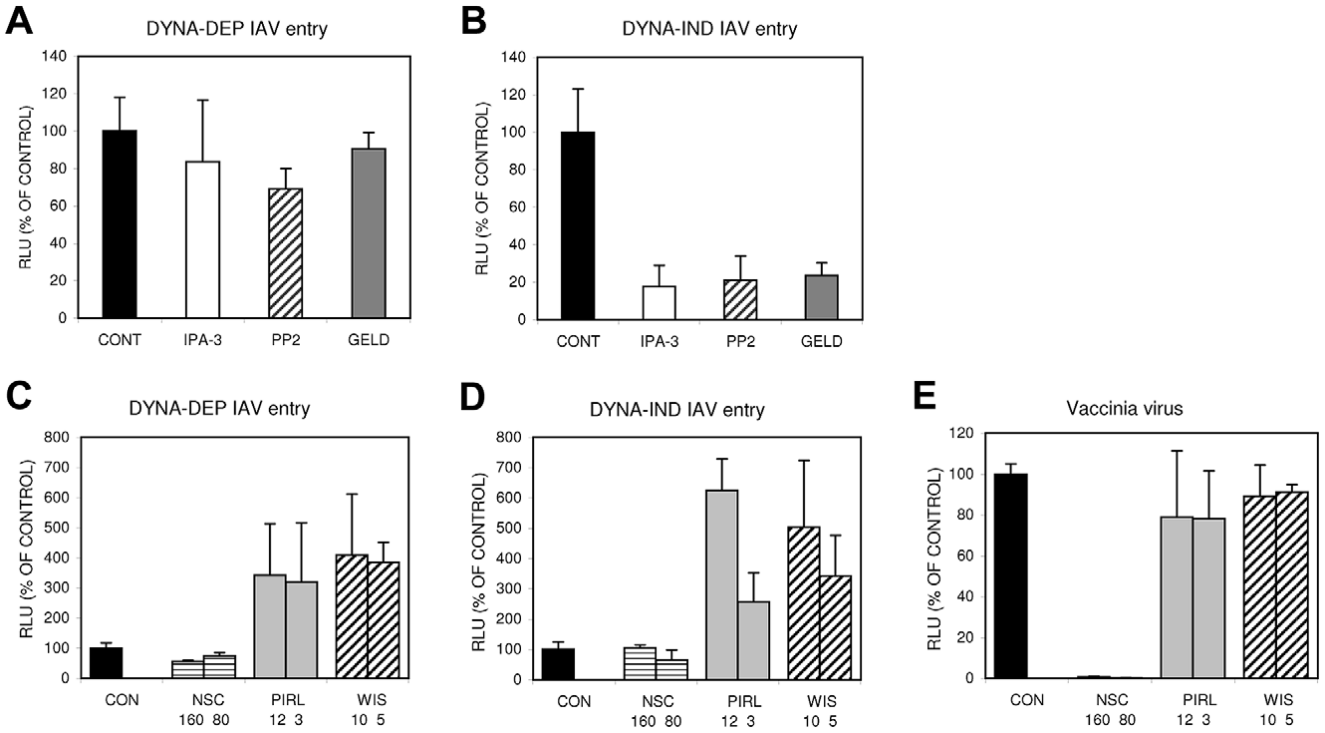
The DYNA-IND IAV entry pathway is sensitive to inhibitors of PAK1, src and HSP90. Inhibitors of PAK1 (40 µM IPA-3), src (10 µM PP2) or HSP90 (5 µM aa-geldanamycin (GELD)) were examined for their effect on DYNA-DEP (A) or DYNA-IND (B) entry using the Gluc-entry assay (HeLa cells; strain WSN; MOI 0.5; incubation with inhibitors from 1 hr prior to infection to 2 hrs p.i.). Inhibitors of Rac1 (80 µM and 160 µM NSC23766 (NSC)), Cdc42 (12.5 µM and 3.125 µM Pirl1) and N-WASP (10 µM and 5 µM wiskostatin (WIS)) were examined for their effect on DYNA-DEP (C) or DYNA-IND (D) entry using the Gluc-entry assay (HeLa cells; strain WSN; MOI 0.5; pre-incubation with inhibitors 1 hr prior to infection) and on infection by vaccinia virus (E). When Wiskostatin, NSC23766 or Pirl1 were added at t = 2 in order to check for their effect on replication we observed a dose-dependent inhibition by Pirl1 (60% inhibition at 12 uM) and no effect by wiskostatin or NSC23766 (result not shown).

Macropinocytosis has been implicated in the entry of several viruses [12], [14]. However, differences in susceptibility to inhibitors suggest that distinct forms of macropinocytosis might be used by different viruses [34], [35]. By screening specific inhibitors in the Gluc-entry assay using DYNA-IND entry conditions we evaluated the possible involvement of a few signaling cascades that have been implicated in the induction of macropinocytosis. Serum-inducible macropinocytosis has been shown to be activated via a myriad of signaling cascades initiated by growth factors binding to transmembrane tyrosine kinase receptors [14], [17], [36], [37], consistent with the results shown in Fig. 5. A prominent downstream effect of these signaling cascades is the activation of p21 associated kinase 1 (PAK1) which in turn can activate a number of different pathways leading to actin network rearrangements that can ultimately lead to the induction of macropinocytosis [38]. Fig. 9A–B shows that 20 µM IPA3, an inhibitor of PAK1 [39], specifically inhibits DYNA-IND entry of IAV. Activation of PAK1 in response to growth factor stimulation often involves upstream signal transduction by members of the Rho sub-family of small GTPases like CDC42 and/or Rac1 [34], [40], [41]. Alternatively, activated CDC42 and Rac1 can induce actin rearrangements independently of PAK1 [34], [40]–[43] by direct interaction with WASP or WAVE family proteins, respectively [44], [45]. However, inhibitors of CDC42 (Pirl1 [46]), Rac1 (NSC23766 [47]) or N-WASP (wiskostatin [48]) did not display inhibitory effects on DYNA-IND or DYNA-DEP entry of IAV (Fig. 9C–D). Instead, Pirl1 and wiskostatin induced a significant, concentration dependent increase of entry. This stimulatory effect was not observed for the control vaccinia virus strain WR, which enters cells via a Rac1-dependent, macropinocytotic pathway [43] (Fig. 9E), indicating that this effect is specific for IAV. The results suggest a requirement for PAK1 in DYNA-IND entry of IAV that does not require activation by either CDC42 or Rac1. Growth factor inducible activation of the tyrosine kinase src has also been linked to the induction of macropinocytosis [49]–[51]; consistent with this observation the src inhibitor PP2 [52] specifically inhibited DYNA-IND entry of IAV (Fig. 9A–B). Remarkably, 17-AA-geldanamycin, a specific inhibitor of the chaperone protein HSP90 [53], also caused specific inhibition of DYNA-IND entry (Fig. 9A–B). HSP90 affects the folding and activity of many proteins but the recent demonstration of direct activation of the catalytic activity of src by HSP90 [54] provides another indication of the involvement of src in DYNA-IND endocytosis of IAV.

In conclusion, like for other viruses utilizing a macropinocytic entry pathway, PAK1 seems to play a crucial role in DYNA-IND entry by IAV. However, this pathway is independent of Rac1 or cdc42 but may require src, either upstream and/or downstream of PAK1.

## Discussion

The data presented in this study demonstrate for the first time that IAV can enter cells via DYNA-IND macropinocytosis in addition to the previously described DYNA-DEP classical CME pathway [1], [2]. Several lines of evidence indicate that the DYNA-IND entry route of IAV that we identified corresponds with macropinocytosis. First of all, the entry pathway is dependent on the presence of serum, a well-known inducer of macropinocytosis. Second, IAV colocalized in vesicles with soluble FITC-dextran, a marker for macropinocytosis. Third, DYNA-IND IAV entry was sensitive to the amiloride-derivative EIPA, the hallmark inhibitor of macropinocytosis [14], [55]–[58]. Fourth, this IAV entry pathway is sensitive to inhibitors or dominant-negative mutants affecting actomyosin dynamics. Fifth, the specific inhibition of DYNA-IND IAV entry by a number of inhibitors of growth factor receptor tyrosine kinases as well as downstream effectors thereof also points at the involvement of macropinocytosis. Finally, macropinocytosis is independent of dynamin [12], [14], [19].

Despite this extensive list of arguments, viral entry by macropinocytosis needs to be considered with caution. The characteristics of the DYNA-IND route of cell entry by IAV are similar, but not identical to the macropinocytic entry routes taken by other viruses, like two different strains of vaccinia virus and by coxsackie virus B [34], [35]. As is shown in Table 1 and discussed in more detail below, the macropinocytic pathways used by each of these viruses have a few unique characteristics. This may very well reflect the growing notion that macropinocytosis represents a number of differentially induced and regulated processes, rather than being a single endocytic pathway [13], [14]. Macropinocytosis has collectively been described as an inducible form of endocytosis by which fluid-phase cargo travels via non-coated, relatively large and heterogeneous organelles that have emanated from extensive protrusions (e.g lamellar ruffles, circular ruffles or retracting blebs) of the plasma membrane [13]. In the case of DYNA-IND IAV entry more extensive studies using electron microscopy will be required to study the morphology of membrane protrusions with which IAV may associate. In addition, live cell imaging microscopy will be required to characterize the exact itinerary that is taken by IAV virions traveling via a macropinocytic process. This is especially important as different routes of IAV entry are likely to converge at some point in the endocytic pathway. Although unlikely, co-localization of IAV particles with fluid-phase dextran as shown in Fig. 8B may thus represent a situation occurring after convergence of several different routes. The use of microscopy to study macropinocytosis is however complicated by the lack of specific membrane-associated markers for any early step of this endocytic process.

**Table 1:** DYNA-IND entry of IAV is similar, but not identical, to macropinocytic entry of other viruses.

	IAV	IAV	VV	VV	CVB
	Dynamin dependent	Dynaminindependent	WR	IHD-J	
**Fluid uptake**					
Induction	NO	YES	YES	YES	
Co-localization	NO	YES	YES		YES
**Cytoskeleton**					
Actin	NO	YES	YES	YES	
Myosins	NO	YES	YES	YES	
**GTPases**					
Rac1	NO	NO	YES	YES/NO	NO
Cdc42	NO	NO	YES/NO	YES	
Rab5	YES	YES	YES	YES	YES
**Kinases**					
PAK1	NO	YES	YES	YES	
PI(3)kinase	YES	YES	YES	NO	
Receptor Tyrosine kinases	NO	YES	YES	YES	
Protein kinase C	YES/NO	YES	YES	YES	YES
SFK (src family kinases)	NO	YES			YES
**Others**					
Na+/H+ exchange	NO	YES	YES	YES	YES
Acidification	YES	YES	YES	NO	NO
Dynamin 2	YES	NO	NO	NO	NO

Data for IAV entry are from this article. Data for vaccinia virus are from [34] and for coxsackie B virus from [35]. Table adapted from [14].

**Figure 10:**
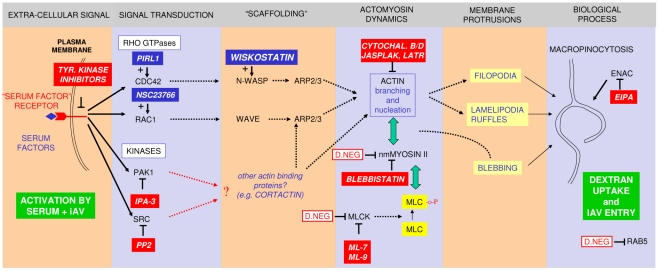
A model for IAV entry by macropinocytosis. The model summarizes the inhibitory (red boxes) or stimulatory (blue boxes) effects of compounds on dynamin-independent IAV entry. The effect of over-expression of dominant-negative mutants is indicated by red-lined boxes. The pathway requires the presence of serum factors in the entry medium and results in the enhanced uptake of dextran and its co-localization with IAV in large vesicles (green boxes). We hypothesize that the interaction of serum factors and/or IAV with receptor tyrosine kinases (RTKs) is the primary signal for the induction of macropinocytosis. A number of RTKs have been shown to be involved in this process in different cell lines. Remarkably, a recently published genome-wide siRNA screen of IAV infection identified the FGF receptor as a host factor required for influenza virus replication [22]. Activation of Rho family GTPases CDC42 and/or Rac1 has been shown to be essential for signal transduction leading to macropinocytosis in many cases [13], [14] but inhibitors are without effect or are stimulatory in the case of IAV entry. Downstream effectors of Rho family GTPases include scaffold proteins like N-WASP and WAVE and protein kinases like PAK1. Macropinocytic entry of IAV however seems to require a Rho family GTPase-independent PAK1 activation mechanism. In addition, src family kinases, which can be directly activated by RTKs, play a role. PAK1 and src have previously been linked to the activation of macropinocytosis via their effect on changes in actomyosin dynamics, a process which is crucial to any form of macropinosome formation [13], [14]. Apart from N-WASP- or WAVE-containing macromolecular assemblies other actin binding proteins can induce such changes (e.g. cortactin, which can be activated by src [81]) and thereby induce the formation of one of the different plasma membrane protrusions that can result in the formation of macropinosomes. In addition to an effect on the formation of plasma membrane protrusions and subsequent macropinosome formation, inhibitors can also affect downstream trafficking and maturation of macropinosomes which might be actin-dependent, but this is not depicted in the scheme.

A model (Fig. 10) based on our results explains the key steps involved in the macropinocytic entry pathway of IAV, which are described in more detail below.

By manipulating the inoculation conditions we were able to experimentally dissect IAV entry into a DYNA-DEP and DYNA-IND route. The DYNA-IND route required the presence of 10% FCS in the entry assay medium. Previously, a strict dependency on a DYNA-DEP entry route for IAV was concluded from experiments with a cell line expressing an inducible dominant-negative mutant of dynamin 2 [59]. In that study, as well as in other entry studies of IAV, entry was performed in DMEM containing 2% serum or BSA. Also in our hands 2.5% serum (Fig. 2A) or 0.2% BSA (result not shown) was not sufficient to allow DYNA-IND entry. We are currently investigating which serum component is responsible for the observed effects on IAV entry. Dialysis of FCS (MW cut off >10 kDa) did not affect its capacity to induce DYNA-IND endocytosis (result not shown), indicating that low molecular weight solutes are not responsible for the observed effect.

Our evidence for a DYNA-DEP and a serum inducible DYNA-IND entry route is based on the use of pharmacological (dynasore, a highly specific inhibitor of dynamin) as well as genetic (siRNA directed against dynamin 2) tools, ruling out the possibility that the inhibitory effect of dynasore was due for instance to absorption of the inhibitor by serum components. Whereas dynasore resulted in near 100% inhibition of DYNA-DEP entry, only 65% inhibition was observed upon siRNA induced silencing of dynamin 2 indicating that the residual levels of dynamin 2 that remain after 48 hrs of silencing still support a low level of DYNA-DEP entry (Fig. 4H). Reversible inhibitors like dynasore [60] offer a major advantage for characterization of IAV entry pathways. They can be applied for a limited period thus preventing the secondary adaptive effects of cells that may occur in response to long-term down regulation of a gene product by genetic methods like siRNA interference. Both entry routes were consistently identified by a viral entry assay quantified by virus induced expression of a luciferase reporter as well as by a VLP entry assay allowing direct analysis of the membrane fusion mediated entry step. The consistent performance of an HA with a strict preference for binding to α2-3 linked sialic acids (from IAV-WSN; our unpublished data) and an HA also binding to α2-6 linked sialic acids (from 1918 IAV [61]) in the VLP entry assay indicates that both pathways can be utilized by HAs of different specificity and may therefore be relevant to avian as well as human IAV infections. Consistently, serum-inducible DYNA-IND entry was observed both in avian DF1 cells and in a human lung epithelial carcinoma cell line A549 (Fig. 3).

The DYNA-DEP and DYNA-IND IAV entry pathways were found by our quantitative assays to be fully redundant. In the presence of serum, the combination of dynasore (inhibiting DYNA-DEP entry) and EIPA (inhibiting DYNA-IND entry) completely abolished entry whereas either drug alone had no effect. EIPA, an inhibitor of plasma membrane Na+/H+ exchangers, has been shown to invariably inhibit macropinocytosis [14], [55]–[58]. As other routes of endocytosis are generally not affected, EIPA is considered as a hallmark inhibitor of macropinocytosis [14], although results obtained with EIPA should be considered with care as long as a mechanistic explanation for its effect on macropinocytosis is not yet fully clear [62]. Occasionally, a moderate two- to three-fold inhibition by dynasore alone was observed (result not shown) indicating that the capacity of the serum-inducible entry pathway is somewhat variable, possibly depending on slight variations in serum quality and factors like cell distribution in the wells that have been reported to influence viral infection [63]. A redundancy in the utilization of CME as well as a clathrin-independent route for entry of IAV has been visualized previously by quantitative live cell imaging [4]. Both routes were operative simultaneously in the same sample and the specific down-regulation of CME did not affect the total number of entry events.

In response to specific extra-cellular signals (e.g. serum induction), changes in the actomyosin network occur that give rise to membrane protrusions required for macropinosome formation [13]. Compounds inhibiting actin polymerization (cytochalasin B and D), depolymerization (jasplakinolide) or sequestering soluble actin (latrunculin A) all specifically inhibited DYNA-IND IAV entry. In addition, the requirement for myosinII activity was established by a specific inhibitor (Blebbistatin) of myosin II ATPase activity and by the expression of a dominant negative mutant of myosinIIA heavy chain. Also, the regulation of myosinII activity by phosphorylation of myosin light chain through the action of MLCK is suggested by the inhibitory effect of MLCK inhibitors ML-7 and ML-9 as well as by the similar effect of an expressed MLCK dominant negative mutant. Recently, a function for the actin cytoskeleton in IAV entry was reported to be required for the entry into polarized epithelial cells but not for entry into non-polarized cells [64]. When using the low-serum conditions used in that paper (2% FCS), we only observed DYNA-DEP entry that was not affected by actin dynamics inhibitors. Perhaps, the polarized cells permit DYNA-IND entry at lower serum concentrations.

The changes in actin network dynamics that can lead to the formation of macropinosomes can be triggered by a number of signaling cascades. Actin dynamics are induced by the activation of growth factor receptor tyrosine kinases by their respective growth factor ligands that are normally present in serum [12]–[14], [17], [36], [37] The signal transduction cascades that link activation of growth factor receptor tyrosine kinases to actin remodeling and macropinocytosis are only beginning to be revealed. The specific inhibition of DYNA-IND entry of IAV by IPA3, an inhibitor of PAK1, provides proof for the involvement of these cascades. PAK1 is a key serine/threonine kinase regulating actin network dynamics but its crucial function in several pathways of endocytosis as well as numerous other cellular processes does not make it a very specific marker [65]. Even so, macropinocytosis has consistently been demonstrated to require PAK1 activation, both in the induction of the process and/or in further downstream trafficking events of macropinosomes [13], [14].

Growth factor dependent activation of PAK1 has most often been demonstrated to depend on upstream activation of small GTPases Rac1 or cdc42 [34], [40], [41]. Different strains of vaccinia virus were recently shown to induce their uptake by macropinocytosis via activation of either Rac1 or cdc42 [34]. Activation of Rac1 has been linked to the induction of macropinocytosis via actin network-mediated formation of lamellipodia and/or circular ruffles whereas cdc42 has most often been implied in the formation of filopodia [44]. An inhibitory effect of the Rac1 inhibitor NSC23766 or the cdc42 inhibitor pirl1 on IAV entry, however, could not be demonstrated. Remarkably, cdc42 inhibitor pirl1 enhanced IAV entry and a similar effect was observed by wiskostatin, an inhibitor of N-WASP which functions directly downstream of cdc42 as a scaffolding complex required for the activation of actin polymerization leading to filopodia formation. Similarly, the macropinocytosis-like entry pathway taken by Coxsackie B virus was also shown to require PAK1 activity that was independent of Rac1 activation [35]. Direct examination of the magnitude and timing of the activation of PAK1 will be required to obtain more insight in the involvement of this complex pathway. The induction of macropinocytosis by a PAK1-dependent mechanism has been associated with ruffling at the cell membrane [12], [14], [15], [37]. The identification of sub-membranous regions with increased actin staining by phalloidin has been interpreted as evidence for ruffling. This was not unambiguously identified by confocal microscopy in the experiments presented in Fig. 8 and Fig. S2 and needs to be investigated in depth by life cell imaging techniques.

In agreement with our observation that the DYNA-IND entry of IAV was inhibited by PP2, an inhibitor of src family kinases, the non-receptor tyrosine kinase c-src has been shown to function as a key signaling intermediate in the induction of macropinocytosis via a mechanism independent of Rac1 or cdc42 [49]–[51]. Downstream effects of c-src on actin networks proceed, amongst others, via phosphorylation of cortactin by c-src resulting in accelerated macropinosome formation [50]. C-src has been shown to associate with macropinosomes [49], [51], both during their formation and their trafficking, while c-src kinase activity is required for macropinocytosis following EGF stimulation of HeLa cells [49]. Interaction of HSP90 with c-src was recently shown to induce c-src kinase activity [54]. Also HSP90 has been demonstrated to associate with macropinosomes, while its specific inhibitor geldanamycin reduced the membrane ruffling that preceded macropinocytosis [66]. Thus, the inhibition of IAV entry via macropinocytosis by AA-geldanamcyin may very well involve the effects of HSP90 on c-src.

As detailed above, the DYNA-IND entry pathway of IAV shares many characteristics with the endocytic pathway macropinocytosis. This is corroborated by the observation that IAV particles and dextran colocalize in large vesicles in the presence of FCS. Several viruses have recently been reported to enter cells via macropinocytosis [12], [14]. Apart from common factors like the requirement for PAK1 activation, actin dynamics and independence of dynamin, virus specific details have been described [34], [35] (Table 1). In part these might be contributed to differences in experimental conditions (e.g. cell types tested) but diversity in the molecular mechanisms by which macropinocytosis can be induced and executed is likely to exist and to be exploited by viruses. Whereas vaccinia virus is able to trigger its own macropinocytic uptake [34], [43], we have described a macropinocytosis pathway that is operational under conditions that are activated by components in serum. Still, this does not exclude signaling induced by virus-host cell interactions, which are for instance suggested by the significant increase of FITC-dextran uptake in the presence of IAV. The possible requirement for co-stimulatory signals from serum components and virus imposes an additional layer of complexity on the analysis of IAV entry via DYNA-IND pathways.

Influenza viruses cause respiratory infections by targeting the epithelial cells lining the respiratory tract. These surfaces are covered by a mucous layer composed of a variety of small solutes and glycoproteins derived among others from goblet cells [67]. This semi-fluid layer in turn conditions the underlying cells and determines their physiological state, including the activities of their uptake and secretion pathways. It will be important to determine to what extent the DYNA-DEP and DYNA-IND IAV entry pathways are operational under the conditions prevailing along the respiratory tract. Current knowledge on the protein composition of the fluids covering the respiratory epithelium is rapidly expanding by the application of proteomic methods to determine the protein composition of bronchial alveolar lavage fluids (BALF). These studies have extended the previous notion that BALF is highly similar in composition to serum. For example, just as for the serum proteome more than 85% of the total protein mass of the BALF proteome is accounted for by albumin, immunoglobulins, transferring, α1-antitrypsin and haptoglobin. In addition, many other proteins have been identified both in serum and in BALF including growth factors that can bind to growth factor receptor tyrosine kinases [68]–[70]. Thus, BALF is likely to harbor, just as serum, the protein factors that can activate signaling pathways that are crucial for the induction of DYNA-IND entry of IAV. In agreement herewith, macropinocytosis has been described as a functional entry pathway of *Haemophilus influenzae* into primary human bronchial epithelial cells [71] although the factors involved in signaling the process have not been identified yet.

In addition to infecting the respiratory tract, IAV has been shown to be able to cause systemic infections involving multiple organs. This has mainly been studied in avian infections [72], [73] or by infection of mice with human-derived H1N1 or H3N2 IAVs [74] but is poorly documented for human infections and may have been underestimated thus far. Obviously, during potential systemic spreading of IAV, the serum-rich conditions that we have demonstrated here to enable the use of alternative entry pathways will be encountered and may contribute to such spreading.

## Material and Methods

### Cells and viruses

MDCK, A549, DF-1 and HeLa cells were maintained in complete Dulbecco's Modified Eagle's Medium (DMEM) (Lonza, Biowittaker) containing 10% (v/v) fetal calf serum (FCS; Bodinco B.V.), 100 U/ml Penicillin, and 100 µg/ml Streptomycin. Chinese Hamster-E36 cells were maintained at 37°C in α-Minimal Essential Medium (Gibco) supplemented with 10% (v/v) FCS, 100 U/ml Penicillin, and 100 µg/ml Streptomycin. Cells were passaged twice weekly. Influenza A/WSN/33 (H1N1) (IAV-WSN) was grown in MDCK cells. Briefly, ∼70% confluent MDCK cells were infected with IAV-WSN at a MOI of 0.02. Supernatant was harvested after 48 hr of incubation at 37°C and cell debris was removed by centrifigutation (10 min at 2000 rpm). Virus was stored at −80°C and virus titers were determined by measuring the TCID_50_ on HeLa cells. The IAV-WSN luciferase pseudovirus (WSN-Ren) system has previously been described [27]. Briefly, WSN-Ren pseudovirus harbors a HA segment in which the HA coding region is replaced by *Renilla* luciferase. The pseudovirus is produced in a MDCK cell line that stably expresses the HA of IAV-WSN. WR-LUC, a firefly luciferase encoding vaccinia virus (strain WR) was previously described [75]. VSV-FL, a firefly luciferase encoding VSV virus was also previously described [76].

### Chemicals

Stocks of bafilomycin A1 (BafA1), dynasore, cytochalasin D, cytochalasin B, Blebbistatin, 17-AA-geldanamycin, ML-7, ML-9, PP-2, 5-(N-ethyl-N-isopropyl)amiloride (EIPA), IPA-3 (all obtained from Sigma-Aldrich), Latrunculin A (Enzo), jasplakinolide, wiskostatin, NSC23766 (all obtained from Calbiochem) and pirl1 (Chembridge) were prepared in dimethylsulfoxide (DMSO). All stocks were stored at −20°C. A kinase inhibitor library composed of 80 kinase inhibitors was obtained from Biomol (2832A[V2.2]).

### Neuraminidase treatment

HeLa cells (10,000 cells/well in 96-well plates) were treated with 2 mUnits of *Vibrio cholerae* neuraminidase (Roche) in 50 µl phosphate-buffered saline (PBS) for 2 hr. After washing with PBS cells were infected with IAV as described.

### Virus-like particle assay

Virus-like particles (VLPs) were produced as described [26]. Briefly, 293T cells were transfected using Lipofectamine 2000 (Invitrogen) with pCAGGS-BlaM1 (encoding a beta-lactamase reporter protein fused to the influenza matrix protein-1), pCAGGS-HA (encoding HA derived from either A/NewYork/1/1918 or IAV-WSN) and pCAGGS-NA (encoding IAV neuraminidase [NA] derived from either A/BrevigMission/1/18 or IAV-WSN) and maintained in OptiMEM. Supernatants were harvested 72 h after transfection and centrifuged to remove debris. VLPs were used for inoculation of cells without further concentration. VLPs were incubated for 30 min at 37°C with trypsin/TPCK for activation of HA. MDCK or HeLa cells grown to near confluency in 24-well plates were inoculated with 250 ul of VLPs after pre-treatment of the cells with inhibitors as indicated. Infection was synchronized by centrifugation at 1500 rpm for 90 min at 4°C and was performed by further incubation at 37°C for 2 h in the absence or presence of 10% FCS and inhibitors as indicated. Detection of beta-lactamase activity was performed as described [25] by loading cells with CCF2-AM substrate (InVitrogen) and subsequent analysis by flow cytometry on a LSRII flow cytometer (Becton Dickinson). Typically 10,000 events were collected and analyzed using FlowJo 8.5.2 software.

### Luciferase assays

The reporter construct pHH-Gluc was derived from plasmid pHH-Fluc [22] by replacing the firefly luciferase coding region with the *Gaussia* luciferase coding region of pGluc-basic (New England Biolabs). Unique SpeI and XbaI restriction sites were introduced into pHH-Fluc using the Quikchange XL Site-directed mutagenesis kit (Stratagene) and oligonucleotides Spe4262 (5-′GCCTTTCTTTATGTTTTTGGCACTAGTCATTTTACCGATGTCACTCAG), Spe4263 (5′-CTGAGTGACATCGGTAAAATGACTAGTGCCAAAAACATAAAGAAAGGC), Xba4260 (5′-GTATTTTTCTTTACAATCTAGACTTTCCGCCCTTCTTGG) and Xba4261 (CCAAGAAGGGCGGAAAGTCTAGATTGTAAAGAAAAATAC). A SpeI site was introduced by site-directed mutagenesis in pGluc-basic directly following the start codon of the *Gaussia* luciferase coding sequence. The unique SpeI – XbaI fragment of pGluc-basic was subsequently cloned into the SpeI-XbaI site of pHH-Fluc resulting in plasmid pHH-Gluc.

Cells were seeded in 96-well plates at a density of 10,000 cells/well and transfected the next day with 10 ng pHH-Gluc using Lipofectamine 2000 (InVitrogen) according to the manufacturer's protocol. After 24 hrs the transfected cells were treated with inhibitors and infected as indicated. At 16 hr p.i. samples from the supernatant were assayed for luciferase activity using the *Renilla* Luciferase Assay system (Promega) according to the manufacturer's instructions, and the relative light units (RLU) were determined with a Berthold Centro LB 960 plate luminometer. WR-LUC and VSV-FL were used to inoculate HeLa cells (10,000 cells/well) at an MOI of 2, in complete Dulbecco's Modified Eagle's Medium (DMEM) (Lonza, Biowittaker). After 7 hr the luciferase activity was detected using the SteadyGlo assay kit (Promega). The addition of 10% (v/v) FCS did not change infection levels for both viruses.

### Confocal immunofluorescence microscopy

Cells were fixed with 3.7% paraformaldehyde (PFA) in PBS and subsequently permeabilized with 0.1% Triton-X-100 in PBS. After blocking with normal goat serum IAV-infected cells were incubated for 1 h with a monoclonal antibody directed against the nucleoprotein (NP) (HB-65; kindly provided by Dr. Ben Peeters). After washing, the cells were incubated with a 1∶400 dilution of Alexa Fluor 488- or 568-labeled goat anti-mouse IgG (Molecular Probes) secondary antibody for 1 h. Nuclei were subsequently stained with TOPRO-3 and after three washing steps, the coverslips were mounted in FluorSave (Calbiochem). Actin was stained using phalloidin labeled with Alexa Fluor 633. The immunofluorescence staining was analyzed using a confocal laser-scanning microscope (Leica TCS SP2). FITC, GFP or Alexa Fluor 488 were excited at 488 nm, Alexa Fluor 568 at 568 nm, and TOPRO-3 at 633 nm.

### FITC-dextran uptake

HeLa cells were grown in 24-well plates on glass coverslips (50,000 cells/well). Prior to FITC-dextran uptake cells were serum-starved for 2 hr in PBS. FITC-dextran (MW70,000, Sigma-Aldrich) was incubated with HeLa cells (final concentration of 0.5 mg/ml) in 500 µl PBS or in PBS containing 10% FCS in the absence or presence of IAV (strain WSN; MOI 10; concentrated and purified by centrifugation through a 15 to 30% sucrose gradient with a 50% sucrose cushion at the bottom) at 37°C. After 15 min cells were washed 4 times with PBS at 4°C, fixed with 3.7% PFA in PBS and subsequently permeabilized with 0.1% Triton-X-100 in PBS. Slides were stained for examination by confocal microscopy as described above. For quantification of FITC-dextran uptake 1.5×10^5^ HeLa cells were infected with IAV-WSN (MOI 10) in suspension in a volume of 1 ml in the presence of FITC-Dextran (1 mg/ml). Infections were performed for 15 min in PBS (containing 2% BSA to reduce unspecific binding of FITC-Dextran) or in PBS containing 10% FCS at 37°C or at 4°C (control for binding of FITC-Dextran to cells in the absence of endocytosis). Mock-infected samples were analysed in parallel. Infection was terminated by addition of 3 ml ice-cold PBS followed by three washes with cold PBS and fixation with 3.7% PFA. 20,000 cells were analyzed by FACS and results were represented as the mean fluorescence which was plotted relative to the uptake in the mock-infection in PBS (after subtraction of background fluorescence obtained at 4°C).

### The effect of dynasore and EIPA on dextran and transferrin uptake

HeLa cells (grown on glass cover slips) were incubated at 4°C for 1 hr with 50 µg/ml Alexa633-labeled Transferin (InVitrogen) in PBS. After 1 hr the medium was replaced by PBS or PBS supplemented with 10% FCS containing IAV (strain WSN; MOI 10) and 0.5 mg/ml FITC-Dextran (Sigma; 70 kDa) and cells were transferred to 37°C for 15 min. After 15 min cells were fixed and stained as described above and examined by confocal microscopy.

### Immunocytochemistry

Cells were fixed with 3.7% PFA in PBS and subsequently permeabilized with 0.1% Triton-X-100 in PBS. Peroxidase was visualized using an AEC substrate kit from Vector Laboratories. IAV-positive cells were detected using bright-field light microscopy.

### Dynamin 2 knockdown

Two siRNA duplexes targeting different sites within the coding sequences of dynamin 2 were obtained from Ambion Inc (15581 (Dynamin 2 siRNA 1) and 146559 (dynamin 2 siRNA2)). A scrambled siRNA (Ambion Inc.) was taken along as a control for non-specific effects of the transfection procedure and was used for normalization. One day after seeding in 96-well plates (6,000 cells/well), the HeLa cells were transfected with a final concentration of 10 nM siRNA using oligofectamine (Invitrogen). 48 h after transfection, the cells were inoculated with the WSN-Ren pseudovirus (MOI 0.5) in PBS or in PBS containing 10% FCS. After 2 h of infection the entry medium was replaced by complete growth medium containing 10 nM BafA1 to prevent further entry. At 16 h post infection intracellular *Renilla* luciferase expression was determined as described above. Each siRNA experiment was performed in triplicate. Cell viability was not affected as determined by performing a Wst-1 cell-viability assay (Roche). Functional knockdown of dynamin 2 mRNA levels was performed by quantitative RT-PCR. using a TaqMan Gene Expression Assay for DNM2 (Hs00191900_m1, Ambion) and using 18S RNA (Hs03928985_g1, Ambion) as a control for normalization. The comparative Ct-method was used for quantification of the results [77]. Reduction of dynamin 2 protein levels was determined by western blotting using polyclonal goat-anti-dynamin 2 C18 (Santa-Cruz SC-6400). A monoclonal against alpha-tubulin (DM1A, Sigma T9026) was used to detect tubulin for normalization. Results were quantified by Densitometric scanning of the dynamin 2 and tubulin signals displayed in Fig. 4H.

### Dominant negative constructs

HeLa cells were grown in 24-well plates on glass coverslips (50,000 cells/well) for 24 hrs. Cells were then transfected (1 µg of DNA with lipofectamine 2000 as described above) with plasmids encoding wild-type or dominant-negative (DN) human MLCK fused to GFP [78], wild-type or DN Rab5 fused to GFP [79], or MyoII-tail or MyoII-head domain fused to GFP [80]. 24 hr after transfection cells were inoculated with IAV-WSN (MOI 1) in PBS or in PBS containing 10% FCS and 80 µM dynasore. 4 hr after infection cells were fixed and stained for examination by confocal microscopy as described above.

### Statistical analysis

An unpaired Student's t-test was used for detemination of statistically significant differences. The use of the term significant in text refers to a comparison of values for which p<0.05.

## Supporting Information

Figure S1 The effect of 80 µM EIPA, 80 µM dynasore (DY) or 80 µM of both inhibitors (DY+EI) on DYNA-DEP (A–D) or DYNA-IND (E–F) entry was examined in the Gluc-entry assay (HeLa cells (A,E); A549 cells (B,F); 293T cells (C,G); CHO K1 cells (D,H); strain WSN; MOI 0.5; incubation with EIPA or DY from −1 hr to 2 hr p.i.). Data were plotted relative to the control (0.2% DMSO).
(3.57 MB TIF)

Figure S2 The effect of EIPA and dynasore on transferrin or dextran uptake in HeLa Cells. (A) Uptake of Alexa633-labeled transferrin (red) in PBS (upper row) or in 10% FCS (second row) in the presence of IAV (MOI 10). (B) Uptake of FITC-labeled dextran (green) in PBS supplemented with 10% FCS in the presence of IAV (MOI 10). Uptake was performed in absence (CONTROL) of inhibitor or in the presence of 80 µM dynasore (DY) or 80 µM EIPA. Contours of the cells are indicated by a gray line. Eight z-stacks of each slide were inspected to assure that transferrin and dextran were inside the cells.
(7.60 MB TIF)
