# Supplementary figures and images for: Dissection of the Influenza A Virus Endocytic Routes Reveals Macropinocytosis as an Alternative Entry Pathway
Source: PLoS Pathog. 2011 Mar 31;7(3):e1001329. doi: 10.1371/journal.ppat.1001329 (PMC3068995; doi:10.1371/journal.ppat.1001329)

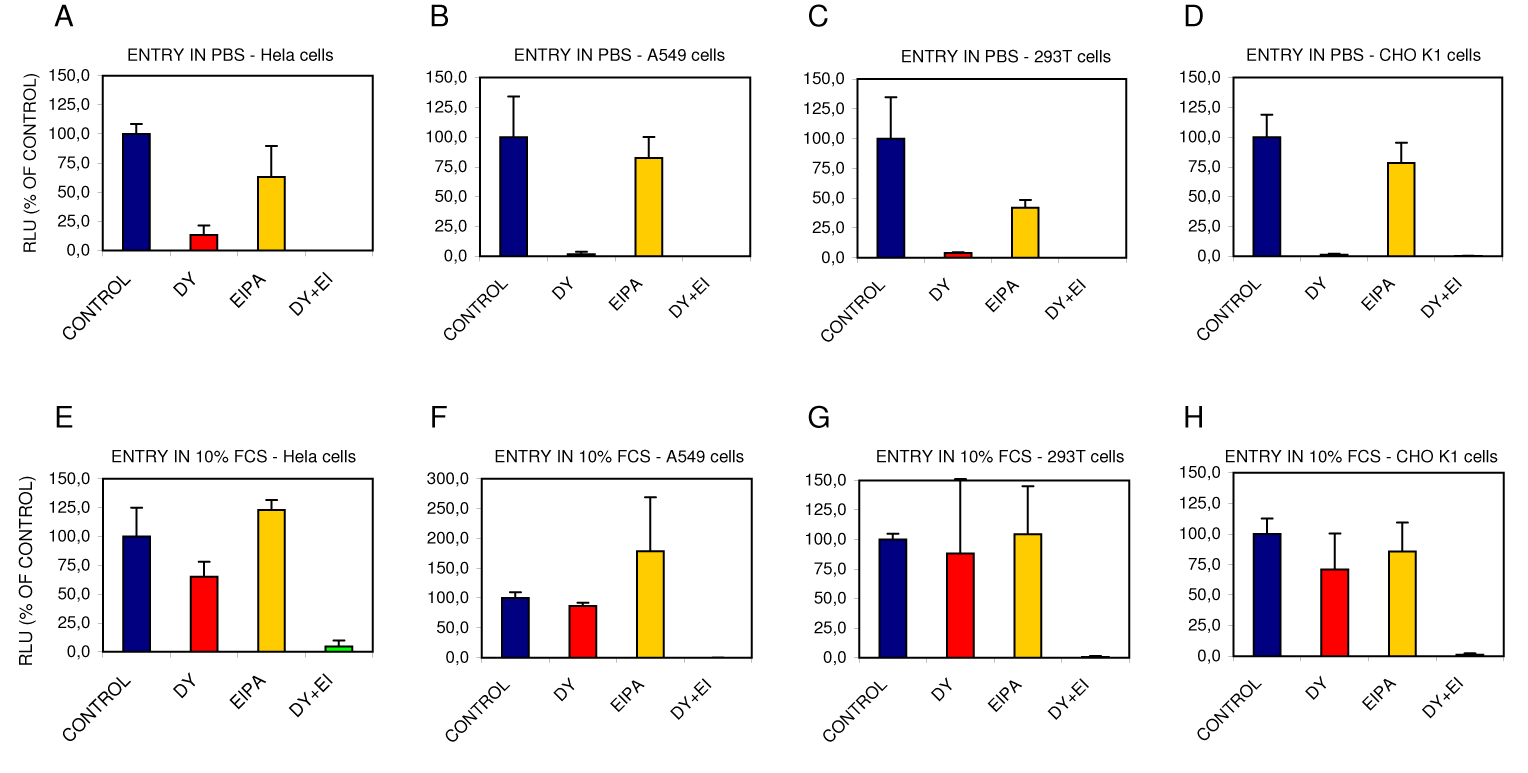

Supplement: Figure S1 — The effect of 80 µM EIPA, 80 µM dynasore (DY) or 80 µM of both inhibitors (DY+EI) on DYNA-DEP (A–D) or DYNA-IND (E–F) entry was examined in the Gluc-entry assay (HeLa cells (A,E); A549 cells (B,F); 293T cells (C,G); CHO K1 cells (D,H); strain WSN; MOI 0.5; incubation with EIPA or DY from −1 hr to 2 hr p.i.). Data were plotted relative to the control (0.2% DMSO). (3.57 MB TIF) [file ppat.1001329.s001.tif]

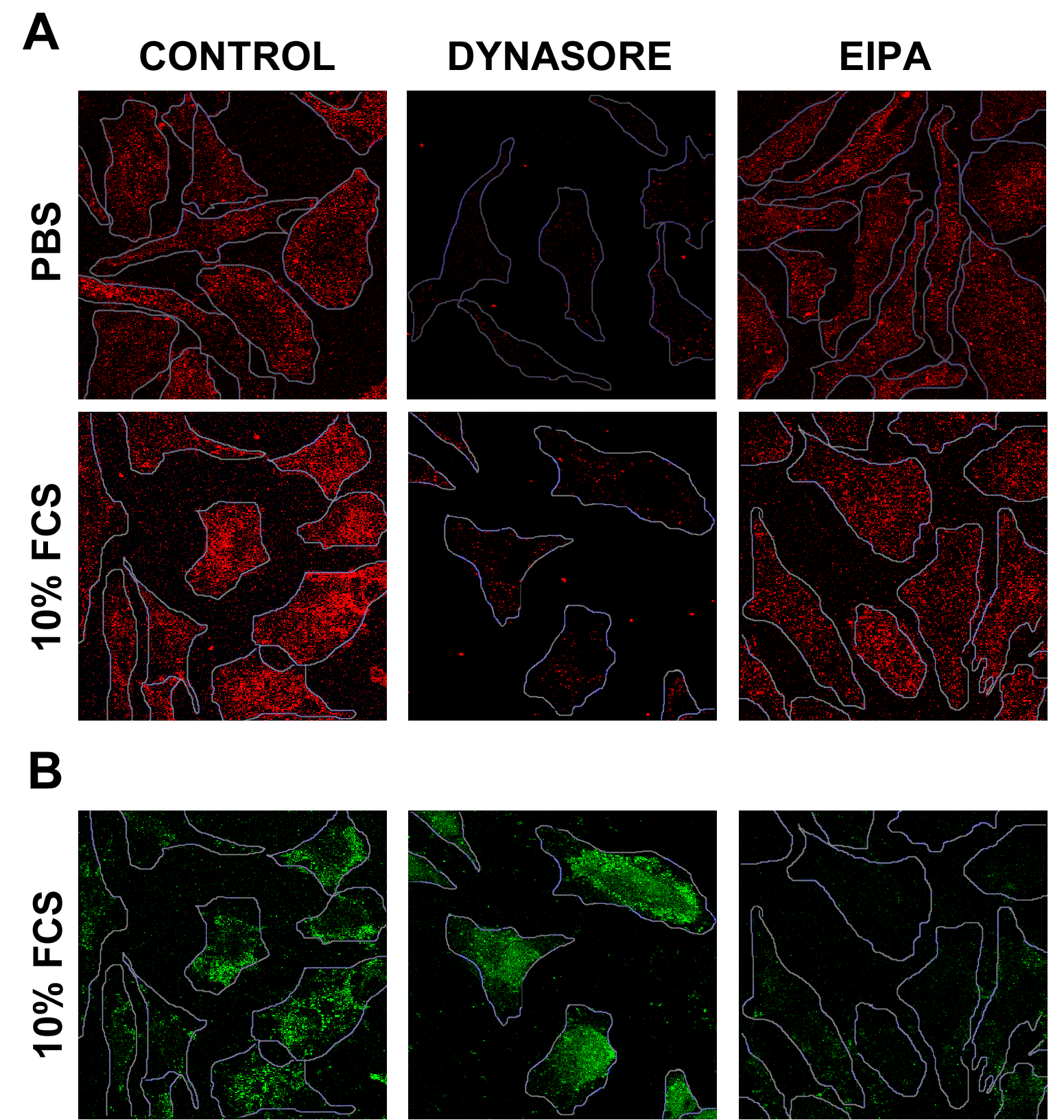

Supplement: Figure S2 — The effect of EIPA and dynasore on transferrin or dextran uptake in HeLa Cells. (A) Uptake of Alexa633-labeled transferrin (red) in PBS (upper row) or in 10% FCS (second row) in the presence of IAV (MOI 10). (B) Uptake of FITC-labeled dextran (green) in PBS supplemented with 10% FCS in the presence of IAV (MOI 10). Uptake was performed in absence (CONTROL) of inhibitor or in the presence of 80 µM dynasore (DY) or 80 µM EIPA. Contours of the cells are indicated by a gray line. Eight z-stacks of each slide were inspected to assure that transferrin and dextran were inside the cells. (7.60 MB TIF) [file ppat.1001329.s002.tif]
